# CRISPR and Beyond: Genome-Editing Strategies in Retinal Stem Cell Research

**DOI:** 10.3390/cells15060489

**Published:** 2026-03-10

**Authors:** Małgorzata Woronkowicz, Maya Natasha Thomas, Sarah Jacqueline Saram, Amanda-Jayne F. Carr, Ana Alonso-Carriazo Fernandez, Zaynab Butt, Piotr Skopiński, Conor M. Ramsden

**Affiliations:** 1 North Devon District Hospital, Royal Devon University Healthcare NHS Foundation Trust, Barnstaple EX31 4JB, UK; malgorzata.woronkowicz.14@alumni.ucl.ac.uk (M.W.); mayanatasha.thomas@nhs.net (M.N.T.); 2 Moorfields Eye Hospital NHS Foundation Trust, London EC1V 2PD, UK; 3 Ministry of Health Singapore, 16 College Road College of Medicine Building, Singapore 169854, Singapore; sarah.saram@mohh.com.sg; 4 Institute of Ophthalmology, University College London, 11–43 Bath Street, London EC1V 9EL, UKana.fernandez.18@ucl.ac.uk (A.A.-C.F.); zaynab.butt.22@ucl.ac.uk (Z.B.); 5 Department of Ophthalmology, SPKSO Ophthalmic University Hospital, Medical University of Warsaw, 00-576 Warsaw, Poland; pskopin@wp.pl; 6 Department of Histology and Embryology, Medical University of Warsaw, 02-004 Warsaw, Poland; 7 Faculty of Health and Life Science, University of Exeter Medical School, Exeter EX1 2HZ, UK; 8 West of England Eye Unit, Royal Devon University Healthcare NHS Foundation Trust, Exeter EX2 5DW, UK

**Keywords:** retina, stem cells, iPSCs, ESCs, CRISPR-Cas9, base editing, prime editing, TALENs, ZFNs

## Abstract

Genome editing has emerged as a transformative approach for understanding and treating retinal degenerative diseases. Combining this technology with pluripotent stem cells provides an ideal platform for modeling human development and disease, and investigating emerging therapeutic strategies ultimately aimed towards in vivo correction. This approach enables both functional studies to understand retinal degeneration and the early development of targeted therapies for inherited disease. This review offers a comprehensive overview of genome-editing techniques and the ability to create new clinically relevant models to understand human disease in retinal research, focusing on the use of the CRISPR-Cas9 system in induced pluripotent stem cells (iPSCs) and embryonic stem cells (ESCs), as well as highlighting recent advancements in base and prime editing. Gene editing in various retinal diseases is discussed in context of studies focusing on disease modeling or developing therapeutic strategies. Continued refinement of these techniques will be essential for advancing translational applications in retinal disease treatment.

## 1. Introduction

The retina is a highly specialized tissue responsible for converting light into neural signals, enabling vision. Retinal degenerative diseases, including age-related macular degeneration (AMD) and inherited retinal dystrophies (IRDs), are among significant causes of irreversible blindness worldwide [1,2]. Genetic mutations that drive inherited disorders may impair the function and survival of essential retinal cell types, such as photoreceptors and retinal pigment epithelium (RPE) cells [2,3]. Current treatment options are limited and often focus on slowing disease progression and addressing secondary effects rather than restoring vision. Stem cell-based approaches, particularly those utilizing pluripotent stem cells, have emerged as promising tools for disease modeling, drug discovery, and regenerative therapies [4,5].

The advent of genome-editing techniques has further expanded the therapeutic potential of stem cells [6]. The Clustered Regularly Interspaced Short Palindromic Repeats (CRISPR)/CRISPR-associated protein 9 (Cas9) system, in particular, has emerged as a powerful and versatile tool for precise gene modifications. Compared to earlier methods such as zinc finger nucleases (ZFNs) and transcription activator-like effector nucleases (TALENs), CRISPR-Cas9 offers greater efficiency and scalability. More recently, advancements such as prime editing and base editing have further refined genome-editing capabilities, allowing for even more precise and predictable modifications without generating double-stranded breaks.

This review outlines the stem cell platforms available for retinal disease modeling and discusses advances in genome-editing techniques, primarily CRISPR-Cas9, to create new models and investigate CRISPR editing as a therapeutic. Studies employing a broad range of stem cell types with the main focus on induced pluripotent stem cells (iPSCs) and embryonic stem cells (ESCs) are discussed in context with individual inherited and degenerative retinal diseases. Current findings on strategies aimed at correcting retinal diseases, enhancing photoreceptor regeneration, and improving transplantation outcomes are presented while highlighting technical challenges, limitations, safety considerations and future prospects.

## 2. Methodology

This comprehensive review was conducted to collate and analyze current advancements in genome-editing techniques, specifically CRISPR-Cas systems, TALENs, ZFNs, prime editing, and base editing, as applied to stem cells in retinal research. A systematic literature search was performed across multiple databases including PubMed, Embase, Scopus, Web of Science, and Google Scholar, covering publications up to March 2025. The search terms combined standardized indexing terms (such as MeSH and Emtree) with free-text keywords, using Boolean operators:

*(“genome editing” OR “CRISPR” OR “TALEN” OR “ZFN” OR “prime editing” OR “base editing”) AND (“retina” OR “retinal cells” OR “retinal organoids”) AND (“stem cells” OR “pluripotent stem cells” OR “iPSC” OR “ESC”)*. Filters were applied for English-language studies and articles involving either human or animal retinal models. Additionally, the reference lists of all included articles were screened manually to identify other relevant studies not captured in the initial database search.

## 3. Retinal Anatomy and Its Contribution to Visual Function

The human retina is a highly active, metabolically demanding neural tissue that extends from the posterior segment of the eye and is a direct continuation of the central nervous system [7]. It functions as the primary photosensitive component of the visual system and converts incident light energy from photons into electrical signals that are processed through a layered neuronal network.

**Figure 1:**
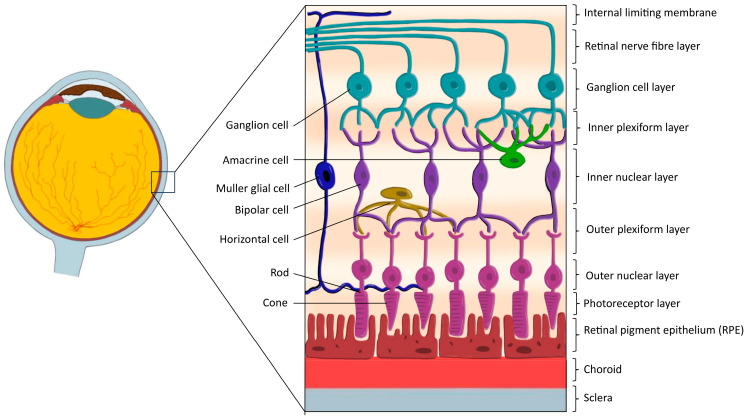
Cross-sectional anatomy of the human eye and retinal organization.

Structurally, the retina consists of ten distinct layers: (1) inner limiting membrane, (2) nerve fiber layer, (3) ganglion cell layer, (4) inner plexiform layer, (5) inner nuclear layer, (6) outer plexiform layer, (7) outer nuclear layer, (8) outer limiting membrane, (9) photoreceptor layer (containing rods and cones), and (10) retinal pigment epithelium (RPE), (Figure 1) [8]. Functionally, these layers can be broadly grouped into photoreceptor, bipolar, and ganglion cell strata, reflecting the vertical organization of visual signal transmission, with synaptic integration occurring within the plexiform layers [9].

The retina contains six major neuronal cell classes—photoreceptors, bipolar cells, horizontal cells, amacrine cells, and retinal ganglion cells—along with Müller glial cells and other supporting glia which together mediate phototransduction, signal modulation, and contrast enhancement [7,9]. Phototransduction is initiated within rod and cone photoreceptors, where photons are converted into bioelectrical signals that are sequentially relayed through bipolar and ganglion cell layers before exiting the eye via the optic nerve [7,8]. This process places exceptional metabolic demands on the outer retina, which is predominantly supplied by the choroidal circulation, while the inner retinal layers receive blood supply from the central retinal artery [9].

The RPE is a monolayer of tightly packed, pigmented epithelial cells located between the photoreceptor layer and Bruch’s membrane, forming a critical interface between the neural retina and the choroid [7,8,10]. Although not a neuronal component of the retina, the RPE is indispensable for retinal homeostasis and maintains the outer blood–retinal barrier through tight junctions, regulates ion and water transport, removes metabolic waste, and phagocytoses shed photoreceptor outer segments in a circadian-dependent manner. In addition, the RPE plays a central role in the visual cycle by recycling retinoids required for photoreceptor function, as photoreceptors themselves lack the capacity to regenerate visual chromophores [9].

Beyond its metabolic and structural roles, the RPE contributes to ocular immune privilege by limiting immune cell access and secreting immunomodulatory factors, including transforming growth factor-β, thereby protecting the retina from inflammatory damage [8,10]. Given the retina’s high oxygen consumption, intricate cellular interdependence, and limited regenerative capacity, dysfunction of photoreceptors or the RPE commonly results in progressive retinal degeneration and irreversible vision loss [8,9].

## 4. Overview of Stem Cells in Retinal Research

**Figure 2:**
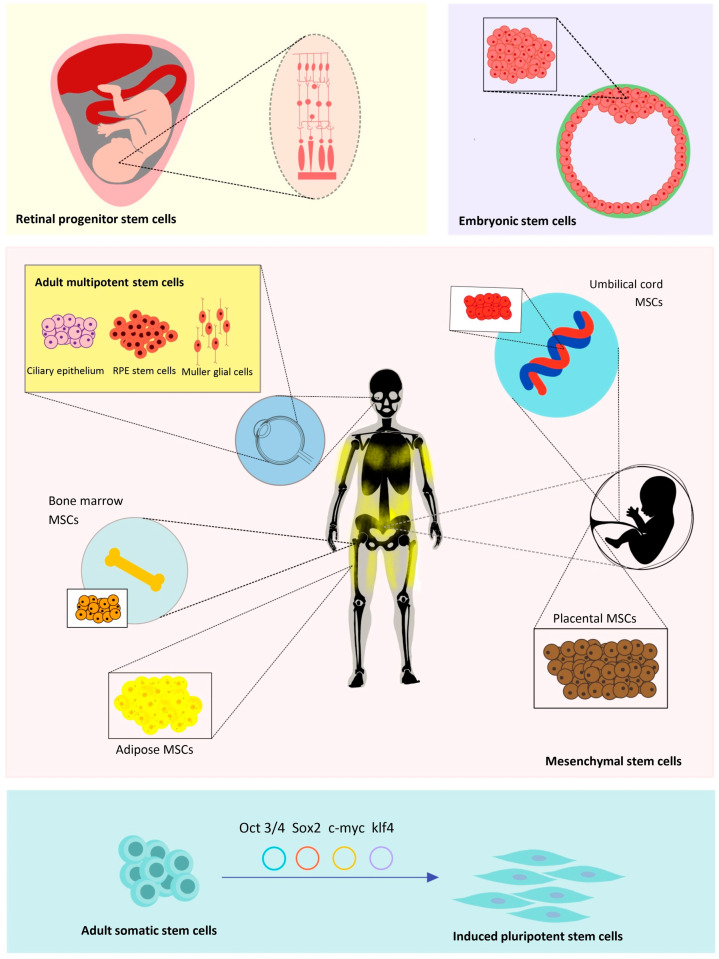
Schematic diagram of stem cells used in retinal research.

Stem cells exhibit several critical characteristics that enhance their therapeutic potential in retinal diseases, including their ability to self-renew indefinitely, their undifferentiated nature, and their capacity to differentiate into specific cell types [11]. Various stem cell types have been identified as promising candidates for stem cell replacement strategies in retinal conditions, and these can be classified into two categories: ‘ocular-derived’ and ‘non-ocular-derived’ stem cells, Figure 2 [9,12].

### 4.1. Ocular-Derived Stem Cells

#### 4.1.1. Retinal Progenitor Cells (RPCs)

Fetal RPCs are multipotent stem cells derived from the fetal retina at 16–20 weeks of gestation which possess the ability to differentiate into multiple retinal cell types within a single lineage [13]. Preclinical studies in animal models have demonstrated that the therapeutic action of transplanted fetal RPCs is mediated through the secretion of neurotrophic factors, which help protect and rescue endogenous photoreceptor cells without causing significant adverse effects [14,15]. In the past decade, several clinical trials (both ongoing and completed) investigating the safety and efficacy of injected human RPC cells in improving BCVA in small cohorts of patients with Retinitis Pigmentosa (RP) have been undertaken. Early results suggest that this may have an acceptable safety profile and could contribute to improvements in visual function; however, whether these benefits can be sustained in the long term and are replicable across a wider cohort remains unproven and there are obvious ethical considerations of sourcing these at scale [16,17,18,19].

#### 4.1.2. Adult Multipotent Stem Cell

Several adult-derived multipotent stem cell populations of neural lineage have been identified in ocular tissues, including ciliary epithelium-derived stem cells, RPE stem cells, and Müller glial cells [20]. These tissue-specific cells have gained attention for their potential in autologous transplantation, allowing ex vivo expansion prior to re-implantation, lowering the chances of immune rejection compared allogenic transplants [20]. Ciliary epithelium-derived stem cells, located at the retinal periphery, can respond to injury in lower vertebrates like zebrafish and amphibians. However, their ability to differentiate into photoreceptors after proliferation in vivo remains unclear [11,20].

The RPE, of neuroepithelial origin, can transdifferentiate in amphibians during early development, though this plasticity declines with maturation [21]. In humans, a small subpopulation of RPE cells retains multipotent potential in vitro, suggesting latent regenerative capabilities [21]. In zebrafish, Müller glial cells function as retinal stem cells, regenerating neural retina under stress [11]. While human Müller cells lack this capacity in vivo, in vitro studies have shown limited differentiation into photoreceptor-like cells under defined conditions, as demonstrated by Gianelli et al. [22]. Furthermore, Lawrence et al. have shown that adult human Müller-derived cell lines display progenitor-like properties, forming neurospheres, expressing neural and photoreceptor markers, and even integrating into host retina in preclinical transplantation models [23]. Notably, in such preclinical models, these grafted cells can also partially restore visual function through the release of neuroprotective factors, despite limited neuronal differentiation [24].

### 4.2. Non-Ocular-Derived Stem Cells

#### 4.2.1. Mesenchymal Stem Cells (MSCs)

Adult MSCs are multipotent stem cells that can be isolated from various tissues, including adipose tissue, placental tissue, bone marrow and the umbilical cord [25]. Clinically, MSCs derived from the bone marrow have been shown to improve vision in patients with non-proliferative diabetic retinopathy and RP [26,27]. Following transplantation, these cells may differentiate into cells with retinal-like characteristics; however, their main role is to provide neuroprotection, reducing immune responses, secreting growth factors and preventing cell apoptosis [28]. Challenges remain in the clinical application of MSCs such as complex isolation and culture requirements, large-scale manufacturing consistency, preservation of functional integrity during storage and transport as well as heterogeneity of isolated MSCs necessitating rigorous standardization and regulatory oversight [25].

#### 4.2.2. Embryonic Stem Cells (ESCs)

ESCs are pluripotent cells derived from the inner cell mass of early-stage embryos, typically obtained from blastocysts [29,30]. Various protocols have been developed to differentiate ESCs into RPE cells for the purpose of replacing diseased cells in retinal degenerative conditions through transplantation [31,32,33]. These techniques have successfully generated ESC-derived RPE cells that closely resemble the morphology and function of endogenous RPE cells, with no reported tumor formation when transplanted into the subretinal space of rodent models of retinitis pigmentosa and AMD [32,34,35]. Clinical trials involving small cohorts of AMD and Stargardt’s disease (SD) patients have demonstrated that ESC-derived RPE cells are safe and well tolerated, particularly when immunosuppressive treatments are used to prevent allograft rejection [36,37,38]. Despite the absence of tumor formation in animal studies, long-term safety data are still lacking, and challenges related to taking immunosuppressive medication long-term and ethical concerns persist [39,40]. Additionally, cultured hPSCs can acquire cancer-associated mutations such as TP53 variants during expansion, underscoring the need for rigorous genomic screening before clinical application [41].

#### 4.2.3. Induced Pluripotent Stem Cells (iPSCs)

IPSCs are pluripotent cells that have been reprogrammed from adult somatic stem cells and can be re-differentiated into various cell types derived from all three germ layers [42]. This technology was first described by Takahashi and Yamanaka in 2006, who identified four transcription factors, Oct3/4, Sox2, c-Myc, and Klf4, vital for reprogramming of mouse fibroblasts into a pluripotent stem cell state [42]. Subsequently, the reprogramming of human somatic cells, such as dermal fibroblasts, was successfully achieved using retroviral transduction with the same transcription factor combination [43]. The discovery of iPSCs offers a theoretically unlimited source of autologous pluripotent cells while also addressing the ethical concerns associated with the use of embryonic stem cells. Somatic cells used for reprogramming can be sourced easily from the patient’s skin, blood or urine [44]. These cells can be cultured, reprogrammed into IPSCs, differentiated into retinal cells, and transplanted back into the same patient without the risk of immune rejection [45].

Genetic alterations and tumorigenesis represent significant concerns in the application of iPSC technology. Previous studies have suggested that retroviral integration in iPSCs may elevate the risk of tumorigenesis, with approximately 20% of mice derived iPSCs developing tumors, potentially due to the reactivation of the oncogene, c-Myc [43,46]. Additional sources of tumorigenic potential include the presence of residual stem cells following differentiation, and intermediate progenitors with proliferative capacity and/or mutated cells [47]. The genomic instability observed in these cells may stem from pre-existing mutations in the parent somatic cells, such as DNA methylation defects or aberrant histone modifications [48]. Furthermore, similar to human embryonic stem cell (hESC) production, iPSCs undergo extended culture periods and repeated passaging, which may increase the risk of chromosomal aberrations and genetic mutations, including cancer-associated TP53 variants that have been detected in several hiPSC lines [41,48].

Significant efforts have been dedicated to addressing the concerns associated with iPSC-based therapies. Key strategies include omitting c-Myc and substituting it with safer, more effective combinations of reprogramming factors, utilizing chemical inductive reprogramming, and purifying differentiated cell populations prior to transplantation [49,50]. Additionally, the implementation of genetic safety mechanisms, such as the introduction of cell apoptosis genes, enables the selective elimination of any residual undifferentiated or tumorigenic cells [49]. While autografts remain the preferred source of therapeutic material, they are time and cost prohibitive to produce from individual patients. In addition, autologous cells may harbour disease causing mutations, particularly in patients with pre-existing genetic conditions, making allogeneic grafts a potentially more viable option [51,52]. Additionally, the development of haplotyped iPSC banks allows selection of lines matching a patient’s HLA haplotype, reducing the risk of immune rejection and potentially minimizing the need for long-term immunosuppressive therapy [53].

### 4.3. Disease Models Derived from Stem Cells

The power of using stem cells to model disease lies in their ability to differentiate into the cells of the chosen organ. To date, this has been most easily achieved using ESCs and iPSCs. In the retina, the major two cell types involved in retinal disease are RPE and photoreceptors. While the RPE, as a monolayer epithelium is relatively easy to culture in isolation in vitro, the photoreceptor cells are best cultured in a three dimensional cluster of cells called retinal organoids (ROs).

#### 4.3.1. Stem Cell-Derived Retinal Pigment Epithelial Cultures

RPE culture from a living donor was first performed in 1996 (a cell line called ARPE-19) and has been a useful model to study ocular diseases [54]. As with many cell lines, with time it has drifted from its original phenotype. The stem cell-derived RPE cells grow as a monolayer, make a barrier, secrete characteristic growth factors, and express RPE specific genes. The discovery that ESC and later iPSC could be differentiated into RPE cells has opened an avenue to retinal research [54].

#### 4.3.2. Retinal Organoids (RO)

RO are three-dimensional, self-organizing structures generated from pluripotent stem cells (PSC), including human ESCs and iPSCs, through stepwise differentiation protocols that recapitulate key aspects of retinal development [55]. Under defined culture conditions, these stem cell-derived tissues can undergo optic vesicle-like morphogenesis and give rise to laminated retinal tissue containing major neuronal and glial cell types, including photoreceptors, bipolar cells, and retinal ganglion cells [56]. Thus, ROs can provide a physiologically relevant in vitro model for studying human retinal development, disease mechanisms, and genome editing approaches, while retaining the genetic background of the originating stem cells [57].

Collectively, pluripotent stem cell-derived retinal cells provide an excellent platform to study genetic eye disease. However, these studies are limited by availability of patient tissues and appropriate controls to understand the impact of inherited mutations. Combining PSCs with genome editing approaches provides a powerful tool to investigate rare mutations and develop isogenic controls to understand diseases. For future gene therapy strategies, PSC provide an ideal platform to test approaches in a diseased cell in vitro.

## 5. Genome Editing Techniques

### 5.1. Zinc Finger Nucleases (ZFNs) and Transcription Activator-like Effector Nucleases (TALENs)

Before the advent of CRISPR-Cas, ZFNs and TALENs were the predominant genome-editing tools, both relying on engineered DNA-binding proteins fused to the FokI nuclease to introduce DSB at specific genomic loci [58]. ZFNs utilize arrays of zinc finger domains that recognize triplet DNA sequences, offering modular but technically challenging customization due to their complex protein–DNA interactions.

TALENs, developed more recently, leverage highly specific TALE domains derived from *Xanthomonas* bacteria, which bind single DNA bases, allowing for more flexible and precise targeting than ZFNs [59]. TALENs differ from ZFNs in that their individual TAL effector proteins, composed of tandem 34-amino-acid repeats, recognize single nucleotides through a simplified recognition code determined by the amino acids at positions 12 and 13, enabling a 1:1 binding ratio and facilitating easier design with improved binding efficiency [60]. However, effective binding typically requires a minimum of twelve TAL units, and the resulting long protein arrays can be cumbersome to use in certain experimental assays.

Although both ZFNs and TALENs demonstrated success in gene editing, their widespread use was hindered by the labor-intensive design and off-target effects. The emergence of CRISPR-Cas9 as a simpler, more efficient, and cost-effective alternative has largely supplanted these earlier technologies, though ZFNs and TALENs remain valuable for specific applications [61].

### 5.2. The CRISPR-Cas System

The CRISPR-Cas system, originally discovered as an adaptive immune mechanism in bacteria and archaea, has revolutionized genome editing in diverse biological fields. The discovery and development of the CRISPR system stem from a series of key observations. In 1987 Yoshizumi Ishino and colleagues first identified Clustered Regularly Interspaced Short Palindromic Repeats (CRISPR) in *Escherichia coli*, though their function remained unknown [62]. Subsequent research by Francisco Mojica et al. expanded the understanding of these sequences, proposing that CRISPR, along with CRISPR-associated (Cas) proteins, functioned as an adaptive immune system in prokaryotes [63]. This hypothesis was experimentally validated by Philippe Horvath and colleagues who demonstrated that CRISPR-Cas provided bacteriophage resistance in *Streptococcus thermophilus* [64]. The field advanced significantly in 2012, when Emmanuelle Charpentier and Jennifer Doudna elucidated the mechanism of the CRISPR-Cas9 system and demonstrated its potential for programmable genome editing [65].

The CRISPR-Cas system utilizes Cas proteins and RNA elements to recognize and cleave specific DNA sequences, thereby enabling precise genetic modifications [65]. Among the various Cas proteins, Cas9 has emerged as the most widely employed nuclease due to its efficiency and programmability. Notably, SpCas9 (*Streptococcus pyogenes* Cas9) has been extensively studied and optimized for genome engineering applications [66]. Subsequently, different orthologs of SpCas9 have been identified in other bacteria including *Staphylococcus aureus*, *Streptococcus thermophiles*, *Neisseria meningitides*, *Francisella novicida* and *Campylobacter jejuni* [67].

The CRISPR-Cas9 system operates through a complex molecular mechanism involving guide RNA (gRNA), which directs Cas9 to a specific genomic locus complex [68]. The gRNA is a synthetic fusion of two naturally occurring RNA molecules: CRISPR RNA (crRNA) and trans-activating CRISPR RNA (tracrRNA). The crRNA contains a sequence complementary to the target DNA site, ensuring specificity, while the tracrRNA interacts with Cas9 to form an active ribonucleoprotein complex. In engineered systems, these two RNA components are combined into a single-guide RNA (sgRNA) to simplify the targeting process. The recognition of target sequences is facilitated by the presence of a Protospacer Adjacent Motif (PAM), a short nucleotide motif required for Cas9 binding, with SpCas9 specifically recognizing the 5′-NGG-3′ motif [69]. Upon PAM recognition, the Cas9-gRNA complex induces a DNA double-strand break at the target site.

**Figure 3:**
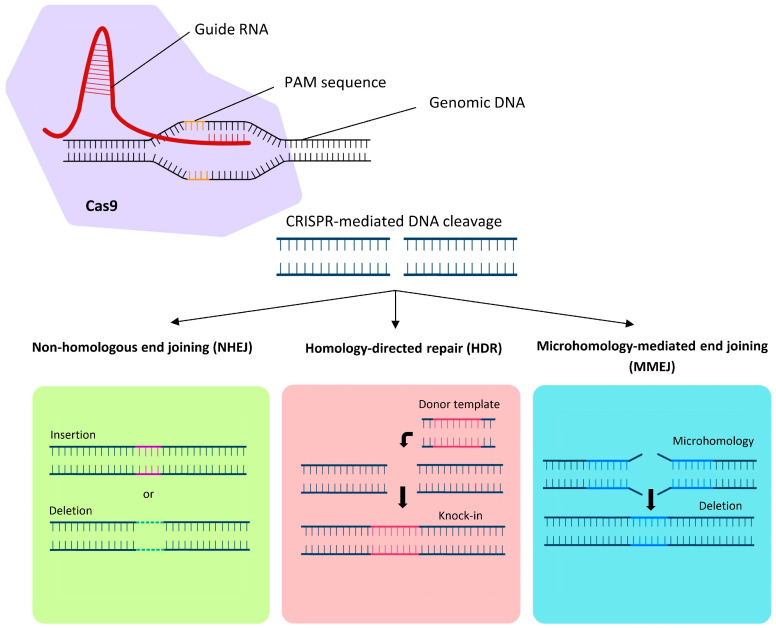
CRISPR-Cas9 genome editing and DNA repair pathways. The figure shows Cas9 creating a double-strand break at a target DNA site adjacent to a PAM sequence. The break can be repaired by non-homologous end joining (NHEJ), microhomology-mediated end joining (MMEJ), or homology-directed repair (HDR), each leading to different genomic outcomes.

The catalytic activity of Cas9 is mediated by two distinct nuclease domains, HNH and RuvC, which cleave the complementary and non-complementary DNA strands, respectively [70]. Following the induction of a double-stranded break (DSB), the cells’ endogenous DNA repair machinery determines the final genomic outcome through one of three main pathways: non-homologous end joining (NHEJ), homology-directed repair (HDR), or microhomology-mediated end joining (MMEJ), (Figure 3). NHEJ, the predominant repair mechanism, is error-prone and often results in insertions or deletions (indels) that can disrupt gene function, a strategy widely used for gene knockout experiments [71]. HDR, in contrast, offers a precise mechanism for genetic modifications by utilizing a homologous DNA template. This method can be used to correct mutations in dividing cells, however the efficiency is significantly reduced in post-mitotic somatic cells, such as those found in the retina. However, modified methods, such as homology-independent targeted integration (HITI), can be particularly useful for gene knock-in approaches, enabling gene insertion even in non-dividing cells [72]. MMEJ, another repair mechanism, relies on microhomology sequences flanking the break site to facilitate small insertions or deletions [73].

CRISPR-Cas systems are broadly classified into two major classes based on the complexity of their effector machinery. Class 1 use multi-subunit protein complexes to target and cleave DNA, while Class 2 relies on single, large multidomain proteins such as Cas9 to achieve this. Each class encompasses several types with distinct target specificities and mechanisms of action [74]. Class 2 systems are more widely used for editing due to their simpler design. Class 2, and a number of Cas nucleases, have been isolated and engineered for use in cells and animal models. New Class 2 nucleases, such as CRISPR Cas12a (previously known as Cpf1) and Cas13 offer great potential for retinal genome editing due to their multiplexing capabilities [75]. Unlike Cas9, Cas12a enzymes require only a single CRISPR RNA (crRNA) and cleave DNA via a RuvC-like endonuclease domain, producing staggered cuts that are favorable for HDR [76]. Cas 13 enzymes target mRNA, offering potential for transient effects, e.g., targeting inflammation.

CRISPR-based technologies have been adapted for precise gene activation (CRISPRa) and repression (CRISPRi), enabling targeted regulation of gene expression without altering the underlying DNA sequence [77,78]. These approaches typically utilize a catalytically inactive dead Cas9 (dCas9), which retains its DNA-binding ability but lacks nuclease activity, preventing double-strand breaks. In CRISPRi, dCas9 is fused to transcriptional repressors, such as Krüppel-associated box (KRAB), to inhibit transcription by blocking RNA polymerase from driving gene transcription or recruiting chromatin-modifying enzymes that induce heterochromatin formation reducing access to gene promoters [78]. Conversely, CRISPRa enhances gene expression by fusing dCas9 to transcriptional activators, such as VP64, with more advanced systems represented by VPR, SunTag, and SAM [77,79,80,81,82].

Delivery of the CRISPR–Cas system involves two principal elements: the cargo and the delivery vehicle. The cargo encompasses the molecular components introduced into cells to mediate genome editing and typically consists of CRISPR-associated elements such as plasmid DNA encoding the Cas9 nuclease and gRNA, mRNA for Cas9 translation combined with gRNA, or preassembled Cas9/gRNA ribonucleoprotein complexes. The delivery vehicles facilitate transport of these cargoes and enable entry into target cells through three primary strategies: physical, viral, and non-viral [83].

Physical delivery approaches use externally applied forces to transiently disrupt cellular and nuclear membranes, enabling intracellular uptake of CRISPR–Cas9 components via mechanisms such as mechanoporation, electroporation, and hydrodynamic injection. While physical methods provide high precision and broad applicability across experimental contexts, their clinical translation is constrained by limited scalability, reduced tissue specificity, and the risk of inducing cellular damage [83].

Most used viral vectors include adeno-associated virus (AAV), lentivirus and adenovirus [84]. Among those, AAV vectors have demonstrated considerable promise due to their low immunogenicity and efficient transduction in retinal tissues [72]. However, AAV’s limited packaging capacity poses challenges for delivering the full-length Cas9 and multiple guide RNAs, prompting interest in dual-vector systems or compact Cas variants like SaCas9 [85], Cas12 [86], or Cas13 [87].

Lentiviral vectors are widely used as delivery systems for CRISPR-based genome editing due to their high transduction efficiency and ability to stably deliver genetic cargo to dividing and non-dividing cells. Their large packaging capacity enables the delivery of Cas nucleases and guide RNAs in a single vector, although concerns remain regarding insertional mutagenesis and prolonged nuclease expression. Consequently, integration-deficient lentiviral vectors and lentivirus-derived virus-like particles are increasingly explored to improve the safety profile of CRISPR delivery [88].

Lentivirus-derived nanoparticles have emerged as promising delivery platforms for CRISPR-based genome editing by utilizing engineered lentiviral structural proteins to package and deliver Cas9, base editors, or prime editors as ribonucleoprotein complexes. This delivery strategy enables transient yet efficient genome editing while substantially reducing the risk of genomic integration associated with conventional lentiviral vectors. By combining receptor-mediated cellular entry with effective intracellular cargo release, these virus-like particles have demonstrated high on-target editing efficiency in vitro and proof-of-concept efficacy in in vivo retinal models, supporting both base editing with reduced bystander effects and precise prime editing with minimal indel formation [89].

Non-viral delivery systems have emerged as a promising alternative to viral vectors by providing enhanced safety profiles and greater flexibility for the delivery of CRISPR/Cas9 components. Biomaterial-based carriers of CRISPR components are generally classified into three main categories: cell-penetrating peptides (CPPs), lipid nanoparticles (LNPs), and biopolymers [90]. They are characterized by their capacity to modulate release kinetics, safeguard therapeutic agents from degradation, and potentially minimize immunogenicity while enhancing cellular uptake. Moreover, synthetic polymer-based delivery systems have been investigated for CRISPR/Cas9 gene editing due to their tunable physicochemical properties, capacity for nucleic acid complexation, and reduced immunogenicity relative to viral vectors [91].

### 5.3. Base Editing

Base editing is an advanced genome-editing technique that enables precise single-nucleotide modifications without introducing DSB, making it a powerful alternative to traditional CRISPR-Cas9-mediated gene editing. It employs a catalytically impaired Cas9 nickase (nCas9) fused to a deaminase enzyme, allowing for targeted conversion of specific DNA bases [92]. The two primary classes of base editors include cytosine base editors (CBEs), which convert cytosine (C) to thymine (T), and adenine base editors (ABEs), which convert adenine (A) to guanine (G), thereby enabling precise correction of point mutations without requiring donor DNA templates or relying on HDR. This method significantly reduces off-target effects and increases efficiency, particularly in non-dividing cells where HDR is inefficient. Although this approach marks a significant advancement in precision gene editing, it does not address insertions, deletions, and frameshift mutations, or offer the flexibility needed to cover all base pair transitions and transversions [93].

### 5.4. Prime Editing

**Figure 4:**
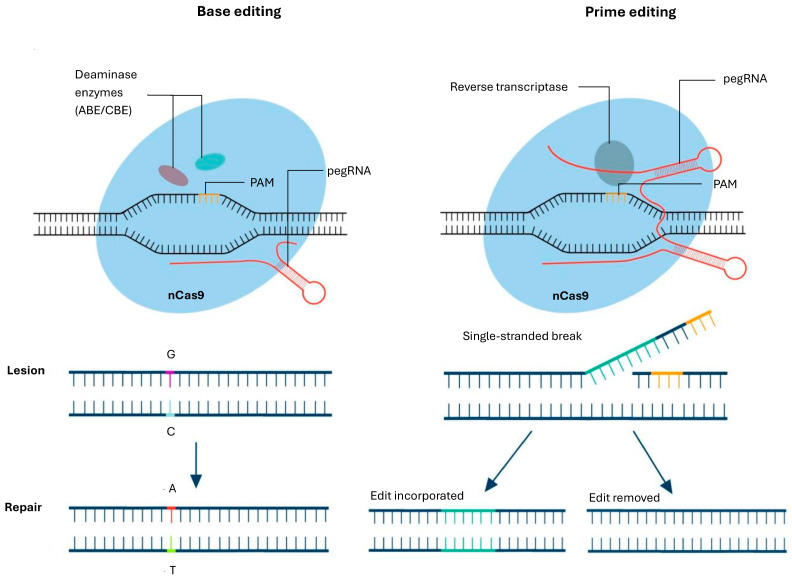
Base- and prime-editing pathways.

Prime editing is a highly precise genome-editing technique that expands the capabilities of CRISPR-based approaches by enabling targeted insertions, deletions, and all possible base substitutions without introducing DSB or relying on donor DNA templates. Developed by Anzalone et al., prime editing utilizes a modified nCas9 fused to a reverse transcriptase (RT) enzyme, along with a specially designed prime editing guide RNA (pegRNA), which encodes both the target sequence and the desired genetic modification, Figure 4, [94]. Once the prime-editing complex is delivered into the cell, nCas9 creates a single-stranded break, allowing the reverse transcriptase to copy the edited sequence directly onto the genome.

This method significantly reduces off-target effects, prevents unwanted insertions or deletions caused by NHEJ, and functions effectively in both dividing and non-dividing cells. Prime editing, despite its versatility and precision, remains limited by factors such as low editing efficiency in certain cell types, challenges in delivering the editing machinery in vivo, and reduced effectiveness in correcting large insertions or complex genomic alterations.

When directly compared, ZFNs and TALENs remain relevant in disease modeling due to their high specificity and fewer off-targets; however, CRISPR-Cas-based systems for modeling and therapeutic development now dominate due to their simplicity and scalability. Although CRISPR can be used to create specific double stranded breaks within the target gene, the post mitotic nature of retinal cells limits potential therapeutic approaches that rely on HDR pathways to correct mutations. Base and prime editing may be particularly suited to retinal applications as many inherited retinal diseases arise from point mutations. Overall, the next generation editors appear more clinically relevant than nuclease-based approaches.

## 6. Genome Editing in iPSCs Models of Retinal Disorders

### 6.1. Age-Related Macular Degeneration (AMD)

AMD is a complex retinal disease that leads to the degeneration of the macula, causing central vision loss [67]. One study focused on the ARMS2 variant in AMD, specifically the A69S (G>T) mutation (rs10490924), which had previously been identified in large GWAS studies as a potential causal factor in AMD [95], (Table 1). CRISPR-Cas9 was used to edit specific single-nucleotide variants (SNVs) within the ARMS2 and HTRA1 genes in patient-specific iPSCs. Functional analysis of edited RPE cells revealed reduced superoxide dismutase activity. Another study explored the role of complement activation in AMD by using CRISPR-Cas9 to knock out C3 in iPSC-RPE cells [96]. The results showed that C3 ablation protected RPE cells from dedifferentiation and epithelial–mesenchymal transition.

Lim et al. identified a novel CFH variant (c.351-2A>G) in a patient with early-onset macular drusen (EOMD) [97]. The CFH gene encodes complement factor H, a key regulator of the immune system’s complement pathway and the FHL-1 protein is a splice variant of the CFH gene [178]. CRISPR-Cas9 was used to introduce this mutation into iPSCs, creating isogenic EOMD RPE cells [97]. Edited iPSC-RPE cells exhibited increased complement activation and membrane attack complex deposition upon exposure to normal human serum, mimicking the local complement dysregulation observed in the disease.

### 6.2. Best Disease (BD)

Best disease (BD), also known as Best vitelliform macular dystrophy (BVMD), is a rare genetic disorder primarily affecting the macula. Mutations in the *BEST1* gene, which encodes the protein Bestrophin-1, are responsible for the condition, with over 250 variants identified to date [67,95]. One study successfully utilized a lentiviral construct to deliver a modified version of the BEST1 gene into RPE cells derived from iPSCs of individuals with the p.Asn296His and p.Arg218Cys mutations [98]. The repaired RPE cells exhibited improved rhodopsin degradation; however, RPE cells with a third mutation, p.Ala146Lys, did not respond to gene augmentation but showed normalization of channel activity following CRISPR-Cas9-based gene editing of the mutant allele. Another study compared CRISPR-Cas9 and TALEN to correct BEST1 c.229C>T mutation [99]. CRISPR-Cas9 was successfully applied in 20% of clones which restored normal chloride channel activity and epithelial permeability in RPE cells, while the use of TALEN was unsuccessful. Another study used an existing human pluripotent stem cell line, H1-iCas9, and supplied gRNA to create a BEST1^-/-^ KO, TMEM16A^-/-^, TMEM16B^-/-^ and LRRC8A^-/-^ knock out human-iPSC RPE cells lines [154]. Authors also introduced point mutations in (I1205T and Y236C) in BEST1 to model gain of function disease.

### 6.3. Retinitis Pigmentosa (RP)

RP is a group of inherited retinal disorders characterized by progressive vision loss, initially affecting night vision and later leading to peripheral vision impairment [179]. The condition primarily targets photoreceptor cells, particularly rods, responsible for vision in low-light conditions, and is caused by mutations in over 100 genes, including RHO, USH2A, RPGR, and PRPH2.

#### 6.3.1. X-Linked Retinitis Pigmentosa (XLRP)

XLRP is a severe form of RP, most often caused by mutations in RPGR or RP2, and typically affects males while female carriers may show variable symptoms, but most become blind by 40 years old [180]. XLRP caused by RP2 mutations, was modeled using CRISPR-Cas9 RP2 knock-out iPSC. Differentiation into retinal organoids (ROs) revealed significant photoreceptor death by day 150, with AAV-based gene therapy restoring rhodopsin expression [100]. Additionally, two studies successfully used HDR-mediated CRISPR-Cas9 to repair the RPGR mutation [101,102]. Single-stranded oligodeoxynucleotides (ssODNs) are short DNA molecules used in CRISPR-based gene editing to repair mutations [181]. They serve as templates for HDR, providing the correct sequence during the DNA repair process, allowing precise insertion or correction of genetic changes at the target site. Bassuk et al. used HDR-mediated CRISPR-Cas9 to repair the iPSCs carrying the RPGR c.3070G>T mutation using an ssODN template [101]. HDR corrected the mutation in 13% of RPGR gene copies which was confirmed using deep sequencing. Another study on XLRP differentiated iPSCs from three patients with c.1685_1686delAT, c.2234_2235delGA, and c.2403_2404delAG mutations in the RPGR gene into RPE cells and ROs [103]. CRISPR-Cas9-mediated repair of the genetic changes led to the restoration of RPE function and structure during long-term differentiation.

#### 6.3.2. Autosomal Dominant Retinitis Pigmentosa (ADRP)

In many cases, ADRP arises from mutations in genes coding for photoreceptor proteins such as rhodopsin (RHO), peripherin (PRPH2), and rod/cone expression factors such as NR2E3, amongst others such as PRPF31 [182].

Liang et al. utilized CRISPR-Cas9 to model PRPF6-related ADRP, editing iPSCs to create the PRPF6 c.2699 G>A mutation [183,184]. The authors initially generated iPSCs from a patient with PRPF6-related ADRP and then differentiated them into RPE cells [183]. Following this, the same research team used CRISPR/Cas9, guided by sgRNA, to correct the *PRPF6* c.2699 G>A mutation, creating an isogenic control [184].

CRISPR/Cas9 has also been used to correct specific *PRPF31* mutations in patient-derived iPSCs carrying pathogenic variants of PRPF31 associated with ADRP (including c.1115_1125del11 and c.522_527+10del16) [108]. Here, the authors generated isogenic controls and created iPSC derived-ROs and RPE models and carried out proteomic analysis on these models’ revealing disruptions in RNA splicing, autophagy, UPR, and visual cycle pathways. In an alternative approach, using a CRISPR-editing to create an iPSC-RPE model of the PRPF31+/− genotype, edited cells were treated with AAV-PRPF31 to test gene augmentation therapy for PRPF31-associated retinal disease [105]. The cells were transduced with AAV2/Anc80 AAP.CASI.V5.PRPF31-mCHERRY.RBG, delivering a functional PRPF31 copy. A CRISPR-Cas9 editing approach has also been used to repair the PRPF31 gene in the iPSCs derived from RP patients carrying the c.709_734dup and c.269_273del mutation [106]. They specifically targeted the PRPF31 gene locus (exon 8), and using a knock-in strategy, inserted a functional copy of the PRPF31 gene which led to normal PRPF31 protein levels in both RPE cells and ROs. Foltz et al. corrected a PRPF8 missense mutation (P2301S) in patients with the RP13 mutation using CRISPR-Cas9 and observed normal differentiation and phagocytic ability in corrected RPE cells [107].

Buskin et al. used CRISPR/ssODN to repair the PRPF31 c.1115_1125del11 mutation in iPSCs and ROs, confirming increased PRPF31 expression and improved cilia length in corrected cells [108]. A study by Diakatou et al. employed CRISPR-Cas9 to knock-out the NR2E2 p.G56R mutation in iPSCs derived from a patient with ADPR. This approach achieved 75% allele-specific knock-out, maintaining normal NR2E3 expression without off-target effects [109]. Using a microfluidic transfection system to co-deliver CRISPR-Cas9 and HDR templates to patient-derived iPSCs, Bohrer et al. were able to use HDR to correct a mutation in the NR2E3 gene [110]. The edited iPSC lines retained normal morphology, karyotype, and pluripotency, and were able to differentiate into ROs. Burnight et al. compared CRISPR-Cas9, both with and without the use of HDR, to correct the Alu insertion in the exon 9 of the MAK gene in iPSCs, showing successful gene correction only with HDR [111].

One group utilized prime editing to correct PRPH2 splice site mutations in patient-derived iPSCs [112]. iPSC lines were generated from PRPH2 patients with the c.828 splice site mutations and differentiated into ROs. Using prime editing, the authors tested a mutation-specific strategy for the prevalent c.828+3A>T mutation and a mutation strategy that targets all c.828 splice site mutations. Both strategies achieved editing efficiencies of approximately 50%. The same group more recently developed a prime editing method to introduce the RPE65 c.1430A>G mutation for ADRP modeling and gene editing [113].

#### 6.3.3. Autosomal Recessive Retinitis Pigmentosa (ARRP)

AARP results from biallelic mutations in genes such as USH2A, MERTK, EYS, and PDE6B and commonly manifests earlier in life, often in individuals without a family history of the disease [185].

CRISPR-Cas9-mediated editing successfully corrected the two most prevalent USH2A mutations, c.2276G>T and c.2299delG, in iPSCs from Usher syndrome patients, restoring normal mRNA levels with no off-target effects [114]. The authors later used these CRISPR-corrected USH2A-iPSCs as isogenic controls in another RO study finding that correction of the USH2A variant c.2276G>T led to enhanced photoreceptor outer segment (POS) formation and partial restoration of the cone photoreceptor phenotype [115].

One study generated a human iPSC line from a patient with late-onset non-syndromic retinitis pigmentosa caused by CLN3 mutations and used CRISPR-Cas9 gene editing to correct one CLN3 variant, creating a co-isogenic control line [116]. A similar study used iPSC derived from a patient with Batten disease, a syndromic lysosomal storage disorder where pathological RP is caused by mutations in the CLN3 gene [117]. The authors used CRISPR-Cas9 to create isogenic control and CLN3 mutant iPSC lines by biallelically deleting exons 7 and 8, allowing them to investigate the mutation’s impact on photoreceptor outer segment phagocytosis.

Mutations in MERTK impair RPE phagocytosis, leading to debris accumulation in the POS and retinal degeneration. CRISPR-Cas9 was used to correct a homozygous frameshift mutation (c.992_993delCA) in iPSCs from a patient with MERTK-associated RP, restoring the phagocytic function in the cells [118]. Moreover, the EYS gene locus encodes a protein involved in photoreceptor cell maintenance and retinal morphogenesis. Recently, dual sgRNA strategy with CRISPR-Cas9 was applied to induce precise deletions of 1,988,210 bp within the EYS gene locus, generating EYSdel iPSC lines to study ARRP and EYS function in ROs [119].

### 6.4. Leber’s Congenital Amaurosis (LCA)

LCA is a rare, inherited retinal disorder characterized by severe vision loss from birth or early childhood [186]. The condition is caused by mutations in over 20 genes, with the most common being RPE65, CEP290, GUCY2D, and CRB1.

Bialleleic pathogenic variants in the LCA5 gene result in a severe form of the disorder, known as LCA5. Athanasiou et al. used CRISPR-Cas9 to generate LCA5 knock-out iPSCs, which were subsequently differentiated into ROs [120]. While LCA5 knock-out did not cause major changes in overall ROs differentiation, significant defects were observed in photoreceptor cilia, specifically in the localization of CEP290 and IFT88 proteins. One study derived iPSCs from a patient with a homozygous nonsense mutation in LCA5 (c.835C>T; p.Q279*) and corrected it using CRISPR-Cas9 [121]. Gene-corrected iPSCs, patient-derived iPSCs, and control iPSCs were differentiated into ROs with misvocalization of opsin and rhodopsin only observed in the patient-derived ROs. Furthermore, gene-corrected ROs exhibited restored lebercilin expression and correct localization to the ciliary axoneme.

ROs differentiated from iPSCs derived from patients with LCA caused by mutations in the CRX gene, known as LCA7 [122]. Two iPSCs lines were differentiated into ROs, carrying either the CRXT155ins4 or CRXK88Q mutation. CRISPR-Cas9 was used to knockout the mutant CRX allele rescuing the photoreceptor phenotype and improving the expression of key markers, including CRX, recoverin, and opsins. Acharya et al. used engineered *Francisella novicida* Cas9 (enFnCas9) variants to correct an LCA2 mutation in patient-specific iPSCs using an adenine base editor [123]. CRISPR-Cas9 has also been applied to insert a red fluorescent protein gene linked with a 2A peptide sequence at the termination codon of the CRX gene in iPSCs to create reporter lines for retinal differentiation studies [124].

LCA type 16 is caused by mutations in the KCNJ13 gene, which encodes Kir7.1, a potassium channel crucial for maintaining proper ionic balance in the retina. Kanzaki et al. used CRISPR-Cas9 to knock out most of the KCNJ13 gene in iPSCs and differentiated them into RPE cells [125]. These KCNJ13-KO iPSC RPE cells demonstrated reduced phagocytosis of POS and misaligned cell structures, suggesting potential pathological molecular mechanisms underlying LCA16. Another study demonstrated the use of silica nanocapsules (SNCs) to deliver adenine base editors to patient-derived iPSC-RPE cells, correcting the KCNJ13 mutation [126].

CRISPR-Cas9 was employed to create an isogenic iPSC line with a frameshift mutation in AIPL1, generating ROs to model LCA type 4 [127]. The model demonstrated that AIPL1 knock-out led to reduced levels of phosphodiesterase 6 (PDE6) and increased cGMP, disrupting phototransduction. Leung et al. found that CRISPR-Cas9 correction of iPSCs with the AIPL mutation restored AIPL1 and PDE6 levels [128]. Another study used CRISPR-Cas9 to create isogenic iPSC lines, one from an LCA4 patient homozygous for c.834G>A, p.W278X and the other from commercially available fibroblasts with a CRISPR-Cas9 AIPL1 knock-out [129]. These iPSCs were differentiated into ROs and utilized to explore AIPL1 gene replacement therapy for LCA4 via AAV-mediated gene delivery.

Mutations in the CEP290 gene can also cause LCA. Burnight et al. targeted a deep intronic mutation, IVS26, in CEP290 using a NHEJ approach, introducing sgRNA sequences flanking the c.2991+1655A>G mutation in patient-derived iPSCs [111]. This strategy successfully deleted the IVS26 mutation, restoring normal CEP290 function. In a separate study, Corral-Serrano et al. generated CEP290 knock-out iPSCs using CRISPR-Cas9 and differentiated them into ROs to evaluate drug therapies, such as the flavonoid eupatilin [130]. Eupatilin treatment rescued key ciliary defects in CEP290-associated retinal models by significantly increasing both cilia incidence and cilia length in patient-derived fibroblasts, CEP290 knock-out cells, and iPSC-derived ROs. In ROs, eupatilin additionally improved photoreceptor outer segment protein trafficking by reducing opsin accumulation in the outer nuclear layer and modulated cilia- and synapse-related transcriptional pathways, supporting a partial functional rescue.

*Crumbs homologue 1* (CRB1) mutations are associated with LCA, RP, and other retinal dystrophies [187]. AAV-based gene augmentation of hCRB1 and hCRB2 in iPSCs from CRB1 mutation patients partially restored RPE morphology and photoreceptor organization [188], while CRISPR-Cas9-mediated correction of the c.2480G>T CRB1 mutation was achieved in another study [131]. Costa et al. utilized prime editing to correct CRB1 mutations in patient-derived iPSC lines by testing 30 combinations of pegRNA and nicking sgRNA for each mutation [132]. With the focus on correcting the prevalent CRB1 mutations p.(Cys948Tyr) and p.(Gly1103Arg), they achieved editing efficiencies up to 72%.

### 6.5. Stargardt’s Disease

Stargardt’s disease is an inherited neurodegenerative condition leading to macular dysfunction and central visual field loss, primarily caused by mutations in the ABCA4 gene. One study used CRISPR-Cas9 to create an ABCA4 knock-out model with iPSC-derived RPE (STGD1-iRPE) from patients with the disease [133]. These STGD1-iRPE cells formed pigmented epithelial monolayers in culture but exhibited disease characteristics, including the accumulation of intracellular lipid and ceramide deposits. In another study, the c.5882G>A (p.Gly1961Glu) mutation, one of the most common mutations associated with Stargardt’s disease, was targeted for correction [134]. Using HDR-mediated CRISPR-Cas9, an iPSC with the ABCA4 c.5882G>A mutation was generated. A split-intein adenine base editing strategy was used to specifically correct the p.Gly1961Glu mutation in the ABCA4 gene. This approach uses the protein trans-splicing mechanism mediated by split inteins to overcome the AAV cargo size limitation. Here, the base editing protein machinery is split in two; each component is then fused to an intein (internal protein segments) within separate AAV vectors. The split protein AAVs are delivered to cells where the base editor is first split, then fused to half of a split intein, and then delivered with in separate AAVs. The base editor is thus reconstituted. This approach was tested in mice and nonhuman primates with the ABCA4 mutation using subretinal injection of AAV. In primates, the AAV-based gene therapy achieved 75% gene editing in cone cells and 87% in RPE cells.

Siles et al. used CRISPR-Cas9 to correct two pathogenic variants of the ABCA4 gene, c.4253+4C>T and c.3211_3212insGT, in patient-derived iPSCs [135]. The authors achieved gene editing via ssODN-mediated repair, targeting two specific mutations which was not replicated when TALEN was employed. Recently, one group used CRISPR-Cas9 combined with C18:1-LAH5 lipopeptide for efficient editing of deep-intronic variants in the ABCA4 gene in iPSC-derived photoreceptor precursor cells, correcting splicing defects and reducing pseudoexon-containing transcripts [136].

Pleiotropy occurs when a single gene influences two or more distinct phenotypic traits. Puertas-Neyra et al. investigated the pleiotropic effects of the c.1354dupT mutation in the PROM1 gene, which causes inherited retinal dystrophies (IRDs) [137]. The study identified three patients homozygous for the c.1354dupT mutation, each exhibiting different phenotypes: cone-rod dystrophy (CORD), RP, and Stargardt’s disease (STG4). The authors generated patient-derived iPSCs from these three patients and then used CRISPR/Cas9 to repair c.1354dupT mutation. The authors successfully corrected the mutation from two patients (CORD and RP) which was confirmed via Sanger sequencing. Although the efficiency of gene correction was low at 10%, this was the first report of a PROM1-related mutation being genetically repaired in patient-derived iPSCs.

### 6.6. Bietti Crystalline Corneoretinal Dystrophy (BCD)

BCD is an autosomal recessive disease that results in progressive retinal degeneration [138]. BCD is primarily caused by mutations in the CYP4V2 gene, which plays a role in lipid metabolism in the retina with approximately 80% of BCD patients carrying mutations in exons 7 to 11.

Li et al., conducted CRISPR-Cas9-mediated gene editing to correct mutations in the CYP4V2 gene in iPSCs derived from BCD patients, aiming to provide insights into potential therapeutic approaches for this condition [189]. The study aimed to investigate BCD iPSC-RPE cells’ sensitivity to blue light-induced oxidative stress. Upon exposure to blue light, BCD iPSCs exhibited a four-fold increase in reactive oxygen species (ROS), more than double the levels of the toxic aldehyde 4-HNE, and a 7.6 times increase in cell death. Correction of the CYP4V2 c.802-8_810del17insGC mutation via CRISPR/Cas9 in an isogenic BCD line restored resistance to blue light damage.

Meng et al. designed a Cas9/sgRNA system to cut at intron 6 of *CYP4V2* and deliver a donor sequence containing exon 7–11 via dual AAV2/8 vectors to BCD patient [138]. The HITI edit restored normal transcription and protein expression, rescued iPSC-derived RPE viability, and improved RPE and photoreceptor structure and metabolism in vivo.

CRISPR-Cas9 edited Cyp4v3 knockout ESCs in C57BL/6J mice showed retinal crystalline deposits and RPE degeneration, providing a model for BCD progression and the application of gene therapy [157].

### 6.7. Sorsby Fundus Dystrophy (SFD)

SFD is a rare autosomal dominant macular degeneration, which presents similarly to AMD, with extracellular deposits beneath the RPE [190]. Engel et al. used CRISPR-Cas9 to correct the S204C TIMP3 mutation in SFD iPSC-RPE cells, reducing sub-RPE deposits [139]. This gene editing mitigated basal laminar and sub-RPE calcium accumulation and improved oxidative stress response in SFD RPE.

### 6.8. ITM2B-Related Retinal Dystrophy

ITM2B-related retinal dystrophy is a rare, autosomal dominant, and slowly progressive retinal degeneration caused by mutations in the ITM2B gene [191]. The condition is characterized by inner retinal involvement, ganglion cell layer abnormalities, and progressive cone photoreceptor dysfunction. Yacoub et al. used CRISPR-Cas9 to correct the ITM2B variant (c.782A>C) in iPSCs via HDR. The cells were transfected with CRISPR components, and successful edits were confirmed by sequencing [140].

### 6.9. Choroideremia

Choroideremia is a rare, X-linked genetic disorder that causes progressive vision loss due to degeneration of the choroid, retina, and RPE [192]. The disease is caused by mutations in the *CHM* gene, which encodes the Rab escort protein (REP1). Raeker et al. utilized CRISPR-Cas9 technology to generate a CHM iPSC-RPE model of the disease by editing the CHM gene to create a knock-out of the REP-1 [143]. This demonstrated the effects of CHM mutations, specifically the under-prenylation of Rab GTPases such as Rab12, which is crucial for autophagy regulation and mTORC1 signaling.

Another study used CRISPR-Cas9 to create two sets of isogenic hiPSC lines: a CHM knock-out line derived from healthy donor cells and a knock-in corrected CHM patient line [144]. REP1 levels were absent in CHM^-^ RPE compared to their CHM^+^ controls. CHM^-^ RPE also exhibited reduced pigmentation and transepithelial electrical resistance compared to controls. All lines formed retinal organoids with prominent photoreceptor structures, showing that early retinal tissue development is not disrupted by CHM mutations.

The disease was also modeled by Iwagawa et al. who introduced a frameshift mutation in the CHM gene of iPSCs [145]. Here, CRISPR-Cas9 system was employed to induce exon-skipping of CHM exon 6 to correct the mutation and improve RPE cell function, particularly under oxidative stress conditions. Of note, AI-based algorithms were used to analyze images of the RPE cells, accurately distinguishing RPE cells with the mutation and those undergoing differentiation without the need for extensive biochemical assays or cell harvesting.

### 6.10. Enhanced S-Cone Syndrome (ESCS)

ESCS is a recessive retinal disorder caused by mutations in the NR2E3 gene [193]. It leads to abnormal photoreceptor development, resulting in an overexpansion of S-cones and impaired rod function. Bohrer et al. used CRISPR-Cas9-based HDR to correct two different NR2E3 mutations in iPSCs derived from two ESCS patients: One patient was homozygous for the c.119-2A>C mutation and the second patient was heterozygous for the p.(Arg73Ser) and p.(Arg311Gln) mutations [146]. Due to the proximity of c.119-2A>C and p.(Arg73Ser), these were targeting with the same HDR and sgRNA agents. The corrected iPSCs were differentiated into retinal cells, demonstrating restored NR2E3 expression and functional recovery. This was demonstrated by the re-emergence of correctly spliced, wild-type NR2E3 transcripts during retinal differentiation of CRISPR-corrected iPSCs, coinciding with the normal developmental window of NR2E3 expression.

### 6.11. X-Linked Juvenile Retinoschisis (XLRS)

XLRS is a genetic condition caused by mutations in the RS1 gene, which encodes retinoschisin, a protein crucial for retinal cell organization and adhesion [147]. RS1 mutations lead to retinal degeneration which is characterized by decreased expression of rod-specific markers (such as NRL) and photoreceptor markers (such as RCVRN). Huang et al. used CRISPR-Cas9 and base editing to investigate XLRS and assess therapeutic effects on photoreceptor development [147]. CRISPR-Cas9 was initially applied to generate isogenic clones by introducing the RS1 C625T mutation in control iPSCs, with a disease phenotype confirmed in IPSC-ROs. Next, base editing was used to precisely repair the C625T mutation, restoring the structural and molecular defects observed in 50% of patient-derived ROs. Another group used CRISPR-Cas9 to introduce the RS1 (c.C304T, p.R102W) mutation into a normal iPSC line, generating an iPSC line (CSUi007-A) without effecting the cells ability to differentiate into germ layers [148].

Mao et al. used CRISPR-Cas9 with a sgRNA and a donor ssODN template to correct the RS1 mutation in iPSCs derived from an XLRS patient. The authors then confirmed correction via sanger sequencing [149].

Nanodiamonds are non-viral carbon based nano-particles which allow for gene editing and delivery. Another study used nanodiamonds to introduce the RS1 c.625C>T mutation into human iPSCs and mice retinas [150].

### 6.12. Hyperornithinemia with Gyrate Atrophy of the Choroid and Retina (HOGA)

HOGA is a recessive inherited disease caused by mutations in the OAT gene, leading to retinal degeneration, muscular atrophy, and blindness. One group used CRISPR-Cas9 to correct the OAT c.1205 T > C mutation in patient-derived iPSCs via HDR, restoring OAT enzyme function, normalizing ornithine levels, and generating corrected isogenic iPSC lines for further study [151].

### 6.13. Ocular Albinism Type 1 (OA1)

OA1 is an X-linked disorder caused by mutations in GPR143, leading to vision impairments, including nystagmus and reduced visual acuity [194]. Torriano et al. targeted an intronic mutation that disrupts normal GPR143 splicing by using CRISPR-Cas12a to correct a point mutation in this gene in OA1 patient-derived iPSCs. The mutation activated a splice site, which was efficiently edited using Cas12a, restoring normal splicing and GPR143 expression, without affecting pluripotency or causing off-target effects [152].

### 6.14. CLCN-Related Retinal Degeneration

CLCN2 is a voltage gated chloride channel protein found in various tissues around the body [195]. CLCN2 mutations effecting ocular tissue is incredibly rare with only five cases documented in the literature. It is a rare autosomal recessive inherited retinal dystrophy caused by biallelic loss-of-function mutations in CLCN2. Xu et al. studied a homozygous c.2257C>T (p.R753X) nonsense mutation in the CLCN2 gene [141]. The authors used CRISPR-Cas9 to repair the CLCN2 mutation in patient-derived iPSC-RPE cells, rescuing chloride channel dysfunction and restoring outer segment phagocytosis.

### 6.15. Late-Onset Retinal Degeneration (L-ORD)

L-ORD is a rare, autosomal dominant maculopathy characterized by the presence of yellow-white punctae, sub-retinal RPE deposits, and geographic atrophy in the macular region, with patients experiencing a continuous decay of their vision at around 40–60 years of age [196]. L-ORD is caused by mutations in the C1QTNF5 gene, with the most frequent cause being the substitution of a serine for arginine in position 163 (p.Ser163Arg) c.489C>G [197]. Five additional mutations in C1QTNF5’s globular C1q domain have also been identified (p.G216C, p.P188T, p.S163R (c.489C>A), p.P186S, and p.S190W) [197].

There is only one example in the literature that has studied CRISPR/Cas9 gene editing of the C1QTNF5 gene [153]. This work established a C1QTNF5 knock-out iPSC line, and two C1QTNF5 isogenic controls using iPSC derived from two patients carrying the S163R mutation. For the knock-out experiments, two guides that target exon 1 of C1QTNF5 were designed and transfected as a plasmid with Cas9, leading to a 107 bp deletion via multiplex NHEJ. Clone screening identified 3 out of 70 clones had biallelic deletion of C1QTNF5, resulting in an in-frame TGA stop codon. For the isogenic control lines, Neiteler targeted the novel spCas9 site formed by the S163R mutation. Both RNP nucleofection and plasmid transfection were studied. While the author comments on the RNP method being inefficient, there was no quantitative analysis of editing efficiency for either method. No off-target editing of the top4/5 loci was observed in the generation of either knockout or isogenic lines. The work continues to explore RPE function in these edited lines. Amongst other results, findings show C1QTNF5 expression is lost in the KO, whereas high molecular weight C1QTNF5 species were similar in WT and isogenic controls, but reduced in mutant lines. Both mutant and KO lines showed increased phagocytosis when compared to WT and isogenic lines. RNAseq revealed mutant lines had upregulated expression of genes associated with extracellular matrix remodeling, lipid metabolism, and cell death, compared to isogenic controls. Finally, mutant iPSC-RPE had increased basal C5b-9 binding compared to isogenic lines, and these colocalized with APOE in drusen-like deposits.

### 6.16. Achromatopsia

Achromatopsia is an autosomal recessive disease which is characterized by an absence of cone function, leading to complete color blindness, photophobia, nystagmus, and reduced vision with CNGA3 and CNGB3-related achromatopsia accounting for 69% of cases [198]. CNGA3 knock-in genetically edited mouse ESCs showed that partial restoration of CNGA3 protein levels could effectively restore cone phototransduction, identifying a therapeutic threshold for gene therapy in CNGA3-associated achromatopsia [158].

### 6.17. Retinoblastoma

Retinoblastoma is a rare, primary intraocular malignancy developing in the retina, primarily affecting children under age 6, usually caused by mutations in the *RB1* tumor suppressor gene [199]. In retinoblastoma modeling, RB1 knock-out ESC-derived ROs displayed disorganization, aberrant differentiation, and a retinoblastoma-like transcriptome [155]. Moreover, cone photoreceptors were identified as the likely cells of origin of retinoblastoma [155]. Additionally, CRISPR-Cas9 was employed to generate heterozygous and homozygous RB1 knock-outs in H1 and H9 hESCs, facilitating the study of retinoblastoma mechanisms and RB1 loss during retinal differentiation [200,201]. A study of *RB1*-deficient CRISPR-Cas9-edited hESCs revealed large teratomas with neural features resembling trilateral retinoblastoma and mitochondrial dysfunction, further characterizing the developmental role of RB1 and demonstrating RB1 null cells’ sensitivity to carboplatin [156].

### 6.18. Optogenics

Recently, Léger-Charnay et al. combined cell therapy with optogenetics, a technique that enables cells to become light-sensitive without requiring the formation of outer segments or connections to the RPE [142]. Using CRISPR-Cas9, they inserted the microbial opsin Jaws, a chloride pump that renders cells sensitive to red light, into the iPSC genome at the AAVS1 locus. These iPSCs can potentially be grafted into animal models of photoreceptor degeneration, aiming to overcome the lack of outer segments or RPE contact which has been a significant barrier to photoreceptor transplant therapy.

### 6.19. Genome Editing in iPSCs and ESCs for Modeling Retinal Disorders

Since the advent of IPSC and the ability to model patient disease in a relevant disease background, IPSC has become the platform of choice. Although hESCs and iPSCs show comparable retinal cell differentiation efficiency, iPSCs are favored for disease modeling due to patient specificity and isogenic controls, while hESCs provide greater genomic stability and manufacturing consistency. RPCs offer lineage commitment but limited scalability. Consequently, most genome-editing studies now rely on iPSC-derived models. Across these studies, several trends emerge. Most applications use editing to create isogenic controls for disease modeling rather than direct therapy. Given the quiescence of IPSC-derived cells, HDR efficiencies remain low in vitro cell culture models, which questions the viability of this as a therapeutic approach, and while base and prime editing show improved precision they are still limited by delivery constraints. Overall, few approaches have progressed beyond proof-of-concept, which suggests that stem-cell editing currently serves predominantly as a modeling tool. However, use of these cells in developing therapeutic editing strategies is increasing as PSCs serve as an ideal pre-clinical tool to assess efficacy in human cells.

## 7. iPSC, RPCs, and hESCs for Modeling Retinal Development

Both iPSCs and ESCs can be genetically engineered to create retinal disease models by generating 3D ROs that closely mimic in vivo retinal structures; iPSCs are particularly appealing because they are patient-specific and avoid the ethical and technical challenges of obtaining ESCs. This approach can also be especially useful for studying the effects of acquired mutations, such as those causing retinoblastoma, although ESCs have likewise been employed in modeling by introducing disease-causing mutations [202,203].

While previous studies have successfully edited terminally differentiated retinal cells, ROs offer the advantage of enabling real-time monitoring of retinal development. Lam et al. employed CRISPR-mediated HDR to generate a triple transgenic reporter iPSC line, named PGP1, which produced ROs expressing all major retinal cell types [204]. The authors utilized CRISPR-Cas9 to insert fluorescent reporter proteins into specific retinal loci (VSX2, BRN3b, and RCVRN) within iPSCs, facilitating the tracking of neural retinal progenitors (NRPs), RGCs, and photoreceptors (PRs) throughout differentiation.

Bai et al. utilized CRISPR-Cas9 to insert an enhanced green fluorescent protein (EGFP) sequence, along with a 2A self-cleaving peptide, into the GNAT2 gene, which is associated with cone photoreceptors [205]. This modification resulted in the creation of a reporter line in which cone photoreceptors (both immature and mature) exclusively expressed EGFP. In ROs derived from these modified iPSCs, the GNAT2-EGFP allele effectively labelled cone photoreceptors from culture day 34 onward.

CRISPR-Cas9 has been used in ESCs to study gene function in retinal development. Creating an NRL-deficient CRISPR-Cas9 edited human ESC line showed that NRL is essential for rod photoreceptor characterization in ROs, offering insights for regenerative retinal therapies [159]. To investigate RB1’s role in early retinal development and tumorigenesis, CRISPR-Cas9-derived RB1-null human ESC ROs were created [161]. RB1 was crucial for retinal progenitor regulation, with its loss leading to increased S-phase entry, apoptosis, and a reduction in retinal cell types, though it did not induce retinoblastoma [160].

In another study, 3D retinal differentiation with CRISPR-Cas9-based gene editing generated Rx-deficient ESCs, confirming that Rx regulates retinal differentiation by promoting neuronal gene expression and suppressing Wnt signaling [161]. While Thrβ2 mutations in mice affect cone subtype specification, CRISPR-Cas9-induced Thrβ2 knock-out in human ESCs demonstrated no significant change in cone subtype ratios, suggesting Thrβ2 is not essential for cone specification in humans [162]. CRISPR-Cas9 was used to create a homozygous knock-out of the CPAMD8 gene in hESCs to study its role in human eye development, due to its connection to the A2M/C3 protein family, which is highly expressed in the lens and retina [163]. The creation of a homozygous *LRP2* knock-out hESC line using CRISPR-Cas9, followed by differentiation into RPE cells, has established a model for studying cellular mechanisms of eye disease associated with *LRP2* deficiency, which leads to RPE dysfunction in *LRP2*-knock-out animals [164].

CRISPR-Cas9 has been used in ESCs to create retinal developmental models. One study generated a CRISPR-Cas9 edited human ESC reporter line which was subsequently differentiated into 3D retinal organoids. These experiments demonstrated that retinal progenitors differentiation towards cone and rod photoreceptors is reliant on PHLDA1 regulation of IGF1 signaling [165]. Another study created a cone photoreceptor reporter line while tagging the GNGT2 gene, enabling tracking of cone differentiation and demonstrating significant migratory activity during differentiation [166]. This provided a useful tool to study human cone development and related diseases [166]. Furthermore, a CRISPR-Cas9-engineered *WDR5B* knock-out and *WDR5B*-FLAG knock-in hESC-derived retinal pigment epithelial (RPE) line was developed, revealing impaired proliferation and elevated cell death with *WDR5B* loss. These findings underscore a vital role for *WDR5B* in maintaining RPE cell viability and suggest functional divergence from its closely related homolog, *WDR5* [167].

A rod photoreceptor reporter line was established by editing the NRL gene, providing a method for exploring rod development and related diseases such as retinitis pigmentosa (RP) [168]. Additionally, CRISPR-Cas9-engineered ESC lines with a selective tdTomato fluorescent reporter under an RGC specific promotor have been used to study RGC development, This system has identified key time points for mechanisms involved in RGC maturation, offering insights for retinal disease therapies [169]. Esmaeili et al. utilized CRISPR-engineered hESC reporter lines differentiated into retinal ganglion cells (RGCs) to profile microRNAs with potential regulatory roles in RGC function. Their findings highlight specific microRNAs that may serve as biomarkers or therapeutic targets in retinal disorders characterized by RGC degeneration [170].

CRISPR-Cas9-edited ESCs have been also used to create allografts without immune rejection in rabbit models for ESC-derived RPE [171]. Here, single and double knockout hESC lines lacking human leukocyte antigens (HLA-I, HLA-II, or both) showed reduced T-cell activation and delayed rejection, while natural killer cell cytotoxicity remained unaffected [171]. This highlights the potential to edit PSC derived cellular therapies prior to transplantation, which may be of importance to allogenic approaches in inherited diseases.

Sluch et al. developed protocols for efficient differentiation of ESCs into RGCs using CRISPR-engineered reporter lines expressing tdTomato and THY1.2, improving RGC differentiation and purification [172]. Moreover, Sluch et al. refined a protocol to successfully generate a high yield of RGCs from hESCs using a fluorescent tagged reporter line [174]. This allowed them to track the differentiation process into RGCs and thereby identify a small molecule called forskolin which, when added early in the differentiation process, increased the percentage of RGCs generated from hESCs [174]. Furthermore, they identified the use of nanoscaffolds to guide axonal growth of RGCs and potentially created a model to study optic nerve injury and regeneration [174]. Another study tracked differentiation of human ESC lines into RPE cells using the fusion of the RPE-specific BEST1 promoter and EGFP gene inserted at the AAVS1 locus using CRISPR-Cas9 [173].

The CRISPR-Cas12a system, which allows targeting multiple genomic loci using several crRNAs from a single transcript, has faced challenges in efficiency, limiting its in vivo applications. Guo et al. developed a hyper-efficient *LbCas12a* variant, hyperCas12a, with the catalytically inactive form, hyperdCas12a, demonstrating significantly improved efficacy for gene activation, especially under low crRNA conditions. The researchers tested this system in postnatal mice in vivo via intravitreal delivery of AAV-hyperCas12a, successfully activating endogenous genes such as Sox2, Klf4, and Oct4, which altered RPC differentiation and enhanced gene repression, while the nuclease-active hyperCas12a enabled more efficient gene editing in retinal ganglion cells [176].

A few studies have explored ZFN and TALEN editing in ESCs and other stem cells. Collin et al. created ZFN-edited human ESCs with a green fluorescent protein at the cone rod homeobox gene, a key retinal development transcription factor, which are useful for isolating and studying photoreceptor precursors during differentiation [175]. Qu et al. developed TALEN-edited Rax::EGFP knock-in rESC lines to investigate the therapeutic potential of ESC-derived RPC transplants in RCS rats [177].

Taken together, the studies discussed here highlight the central role of iPSCs in modeling human retinal development. The authors note that although both hESCs and iPSCs are capable of retinal differentiation and precise genome editing, hESCs have been preferentially utilized in developmental studies, where reproducibility and controlled lineage specification are essential. In contrast, iPSCs dominate disease modeling due to their patient specificity and capacity to generate mutation-matched isogenic controls. This is not limited to retinal studies, with large-scale bibliometric analyses demonstrating this same trend in iPSC research [206,207].

## 8. Conclusions and Future Directions

Genome-editing technologies have rapidly transformed the field of retinal research, offering unprecedented opportunities to model, understand, and potentially correct a wide range of retinal diseases. CRISPR-Cas9, in particular, has emerged as a leading platform due to its relative ease of design, robust editing efficiency, and adaptability across various stem cell systems. The application of this technology in induced pluripotent stem cells (iPSCs) and embryonic stem cells (ESCs) has enabled the generation of disease-relevant retinal models, facilitated targeted gene corrections, and supported preclinical investigations into cell replacement therapies.

Despite these promising advances, several challenges remain when developing therapeutic gene-editing strategies. While delivery to cells-in-a-dish models are successful, the efficient and cell-type-specific delivery of genome-editing components to retinal cells, especially in vivo, remains a significant barrier. AAV vectors are commonly used in retinal gene therapy but are limited by cargo size, which is particularly problematic for prime editors and some base editors. In addition, achieving uniform distribution across relevant retinal layers while avoiding off-target transduction of non-target cells is challenging. Strategies to address these issues include the development of dual- or split-AAV systems, engineered viral capsids with enhanced retinal tropism, and non-viral delivery methods such as lipid nanoparticles or electroporation. Subretinal or intravitreal delivery routes can also be optimized depending on the targeted retinal cell population [208,209].

A major challenge for in vivo therapeutic applications of CRISPR-, prime-edited, or base-edited stem cells in retinal research is achieving sufficiently high editing efficiency while maintaining precision in post-mitotic cells. Moreover, base and prime editors have sequence-context constraints that limit the range of pathogenic variants that can be efficiently targeted. Potential strategies to overcome these obstacles include optimizing editor variants with enhanced activity in non-dividing cells, using retina-specific promoters to improve expression, and applying computational guide RNA design to maximize on-target efficiency. Advances in editor engineering, such as smaller and more processive prime editors, may further improve applicability in retinal tissues [94,209].

Off-target editing and unintended genomic alterations represent a critical concern for the clinical translation of CRISPR-, prime-, and base-edited stem cells in retinal applications. Base editors can induce bystander mutations within the editing window, while prime editors may generate low-frequency insertions or deletions at off-target sites [210]. Several strategies have been described to reduce the risk including engineering of high-fidelity Cas9 variants [211,212], use of truncated gRNAs [213], delivery of Cas9 as protein–RNA complexes [214], chemical modification of sgRNAs [215], and application of artificial intelligence to optimize gRNA design [216,217,218]. Comprehensive in vivo off-target assessment is also essential before clinical application [219,220].

Another major challenge is ensuring the survival, immune compatibility, and functional integration of edited stem cells within the retinal tissue. Immune responses against Cas proteins, viral vectors, or edited cells can reduce therapeutic efficacy and cause inflammation that damages the retina [221,222]. Strategies to overcome these challenges include using immune-evasive editor variants and transient or inducible editing systems [223]. Combining genome editing with supportive biomaterials or neurotrophic factors may further enhance cell survival and integration in the retinal microenvironment [223].

Ethical considerations surrounding the use of ESCs, and the regulatory complexities associated with genome-edited products, further complicate clinical translation. These challenges may be mitigated by prioritizing ethically acceptable cell sources such as induced pluripotent stem cells and by maintaining transparent regulatory frameworks that emphasize patient safety and ethical oversight [224].

The potential to use stem cells for autologous therapies in retinal disease is especially compelling when paired with precise genome-editing tools such as CRISPR. In this approach, a patient’s own cells could be reprogrammed into retinal cell types, corrected for disease-causing mutations, and then transplanted back without the risk of immune rejection. Such strategies could address the underlying genetic cause of inherited retinal disorders rather than only slowing disease progression and might represent a promising path towards personalized treatments.

In conclusion, while considerable progress has been made in harnessing genome-editing tools within stem cell platforms for retinal therapy, continued multidisciplinary efforts are essential to overcome current limitations and unlock their full therapeutic potential.

## Figures and Tables

**Table 1:** Summary of genetic targets in stem cells for retinal research using various gene-editing techniques.

Ref.	Genetic Target	Outcome	Gene-Editing Technique
[95]	ARMS2 p.A69S—Therapeutic approach	CRISPR-Cas9 was used to edit specific single-nucleotide variants (SNVs) within the ARMS2 and HTRA1. Functional analysis of edited RPE cells revealed reduced superoxide dismutase activity.	CRISPR-Cas9 (HDR-iPSC)
[96]	C3—Disease modeling	C3 knock out in healthy iPSCs for disease modeling. The results showed that C3 ablation protected RPE cells from dedifferentiation and epithelial-mesenchymal transition.	CRISPR-Cas9 (NHEJ-iPSC)
[97]	CFH (c.351-2A>G)—Disease modeling	CFH variant (c.351-2A>6) knock in for disease modeling. They reported that editing resulted in restored CFH/FHL-1, complement overaction was mitigated and MAC deposition was reduced.	CRISPR-Cas9 (HDR-iPSC)
[98]	BEST1 p.N296H and p.R218C—Therapeutic approach	CaCC chloride channel activity (lost in BEST1 disease) was fully restored in all edited adBD iPSC-RPE shown with patch-clamp electrophysiology.	CRISPR-Cas9 (NHEJ-iPSC)
[99]	BEST1 c.229C>T—Therapeutic approach	Gene correction was achieved in 20% of clones.	ssODN mediated HDR-iPSC
[100]	RP2 c.358C>T (p.R120X)—Disease modeling	RP2 gene knock out for disease modeling. Knock out resulted in significant photoreceptor death by day 150, with AAV-based gene therapy restoring rhodopsin expression.	CRISPR-Cas9 (NHEJ-iPSC)
[101]	RPGR c.3070G>T—Therapeutic approach	Correction of gene mutation in 13% of gene copies.	CRISPR-Cas9 (HDR-iPSC)
[102]	RPGR exon 14—Therapeutic approach	Gene correction of RPGR in iPSC. Photoreceptor structure and cilia length were rescued in RO upon editing.	CRISPR-Cas9 (HDR-iPSC)
[103]	RPGR c.1685_1686delAT (exon 14)—Therapeutic approach	Gene correction of RPGR in patient iPSCs. Correction resulted in restoration of normal photoreceptor development, electrophysiology, and cilia formation.	CRISPR-Cas9 (HDR-iPSC)
[104]	PRPF31 c.1115_1125del11 and c.522_527+10del16—Disease modeling	Gene correction in patient iPSCs to create isogenic control for disease modeling. Comparison revealed uncorrected iPSCs to exhibit disruptions in RNA splicing, autophagy, UPR, and visual cycle pathways.	CRISPR-Cas9 (HDR-iPSC)
[105]	PRPF31—Disease modeling	Knock in of PRPF31 into wild-type iPSC to generate disease model to test gene augmentation therapy.	CRISPR-Cas9 (NHEJ-iPSC)
[106]	PRPF31 p.C247—Therapeutic approach	Knock in of functional PRPF31 copy to correct gene. Editing led to normal PRPF31 protein levels in both RPE cells and ROs.	CRISPR-Cas9 (HDR-iPSC)
[107]	PRPF8 c.6901C>T—Therapeutic approach	Gene correction of diseased iPSCs and creation of isogenic control. Normal differentiation and phagocytic ability was observed in corrected RPE cells.	CRISPR-Cas9 (HDR-iPSC)
[108]	PRPF31 c.1115_1125del11—Therapeutic approach	To validate the function of PRPF31. Results showed increased PRPF31 expression and improved cilia length in corrected cells.	CRISPR-Cas9 (HDR-iPSC)
[109]	NR2E3 c.166G>A (p.G56R)—Therapeutic approach	G56R allele-specific knockout for gene correction achieving 75% allele-specific knockout without off-target effects and maintaining normal NR2E3 expression.	CRISPR-Cas9 (NHEJ-iPSC)
[110]	NR2E3 c.119-2A>C—Therapeutic approach	Gene correction with corrected iPSC lines retaining normal morphology, karyotype, and pluripotency, and were able to differentiate into ROs.	CRISPR-Cas9 (HDR-iPSC)
[111]	MAK: 353-bp Alu insertion in exon 9 CEP290: c.2991+1655 A>G RHO: c.163 C>A (p.P23H) Therapeutic approach	Gene correction successful only with HDR. Gene correction successful with deletion of the IVS26 mutation, restoring normal CEP290 function.	CRISPR-Cas9 (HDR-iPSC)
[112]	PRPH2 c.828+1G>A—Therapeutic approach	Gene correction with editing efficiencies of approximately 50%.	Prime editing (iPSC)
[113]	RPE65 c.1430A>G—Disease modeling	Knock-in prime editing for ADRP disease modeling.	Prime editing (iPSC)
[114]	USHA2A c.2276G>T and c.2299delG—Therapeutic approach	CRISPR knock-out editing of patient iPSCs restoring normal mRNA levels with no off-target effects.	CRISPR-Cas9 (HDR-iPSC)
[115]	USH2A-RP c.2276G>T and USH2A-USH-1 c.2299delG (USH)—Therapeutic approach	Correction of the USH2A variant c.2276G>T led to enhanced photoreceptor outer segment (POS) formation and partial restoration of the cone photoreceptor phenotype.	CRISPR-Cas9 (HDR-iPSC)
[116]	CLN3 c.175G>A—Disease modeling	Creation of isogenic control for disease modeling.	CRISPR-Cas9 (HDR-iPSC)
[117]	*CLN3^Δ7–8/Δ7–8^*—Disease modeling	Creation of isogenic control and CLN3 mutant line for disease modeling.	CRISPR-Cas9 (NHEJ-iPSC)
[118]	MERTK c.992_993delCA—Disease modeling	Creation of isogenic lines with heterozygous and homozygous correction of mutation in patient iPSCs for disease modeling. Restoration of the phagocytic function in the cells was noted.	CRISPR-Cas9 (HDR-iPSC)
[119]	EYSdel—Disease modeling	Successful creation of the EYSdel iPSC model by gene knock-out.	CRISPR-Cas9 (NHEJ-iPSC)
[120]	LCA5 c.291_291delAT and c.291delT—Disease modeling	Gene knock-out in patient iPSCs to create a disease model. Edited cell lines differentiated into ROs but significant defects were observed in photoreceptor cilia, specifically in the localization of CEP290 and IFT88 proteins.	CRISPR-Cas9 (NHEJ-iPSC)
[121]	LCA5 c.835C>T (p.Q279)—Disease modeling	Correction of diseased iPSCs to create a disease model. Gene-corrected organoids exhibited restored lebercilin expression and correct localization to the ciliary axoneme.	CRISPR-Cas9 (HDR-iPSC)
[122]	CRX (CRXT155ins4 or CRXK88Q)—Disease modeling	Knock-out of mutation in diseased iPSCs to create a disease model. The study showed rescue of the photoreceptor phenotype and improved expression of key markers, including CRX, recoverin, and opsins.	CRISPR-Cas9 (NHEJ-iPSC)
[123]	RPE65 c.992G>A (p.W331)—Therapeutic approach	Single base correction successfully restored RPE65 sequence with successful differentiation into RPE.	Base editing with enFnCas9 (iPSC)
[124]	3′ end of the *CRX* gene—Therapeutic approach	Knock of 2A–E2-Crimson RFP reporter at the 3′ end of the CRX gene to create an endogenous reporter iPSC line in which CRX-expressing photoreceptor precursors fluoresce.	CRISPR-Cas9 (HDR-iPSC)
[125]	KCNJ13 exon 2 and 3—Disease modeling	Knock-out of exon 2 and 3 of KCNJ13 from healthy iPSCs to create a disease model Phagocytosis of POS and misaligned cell structures was noted in the edited cells.	CRISPR-Cas9 (NHEJ-iPSC)
[126]	KCNJ13 W53—Therapeutic approach	Base editing partially restored Kir7.1 function in iPSC-RPE cells; a subset of cells exhibited full functional rescue and low efficiency outcomes of Cas9.	Both ABE Base editing with SNCs and CRISPR/Cas9 (HDR-iPSC) were employed
[127]	AIPL1 exon 1—Disease modeling	AIPL1 knock-out to create isogenic controls for disease modeling. The study noted that AIPL1 knockout led to reduced levels of PDE6 and increased cGMP.	CRISPR-Cas9 (NHEJ-iPSC)
[128]	AIPL1 c.834G>A, p.W278X—Therapeutic approach	Correction of AIPL1 nonsense mutation in iPSCs to create isogenic control. Gene correction restored AIPL1 and PDE6 levels.	CRISPR-Cas9 (HDR-iPSC)
[129]	(1) AIPL1—Disease modeling	(1) AIPL knock-out to create a disease model.	(1) CRISPR-Cas9 (NHEJ-iPSC)
	(2) AIPL c.834G>A, p.W278—Therapeutic approach	(2) Gene correction of patient iPSCs to create an isogenic control. These lines were utilized to explore AIPL1 gene replacement therapy for LCA4 via AAV-mediated gene delivery.	(2) CRISPR-Cas9 (HDR-iPSC)
[130]	CEP290 exon 6—Therapeutic approach	Generation of CEP290 knock-out lines for drug trials e.g., eupatilin.	CRISPR-Cas9 (NHEJ-iPSC)
[131]	CRB1 c.2480G>T—Therapeutic approach	Creation of corrected iPSCs as a genetically defined control for studying CRB1-related RP in retinal organoids.	CRISPR-Cas9 (HDR-iPSC)
[132]	CRB1 p.C948Y and p.G1103R—Therapeutic approach	Gene correction efficiency up to 72%.	Prime editing (iPSC)
[133]	ABCA4 c.6088C>T—Disease modeling	ABCA4 knock-out to create a disease model from patient iPSCs. Corrected cells differentiated normally but exhibited disease characteristics.	CRISPR-Cas9 (NHEJ-iPSC)
[134]	ABCA4 c.5882G>A—Disease modeling	CRISPR-Cas9 HDR was used to generate a disease model which was corrected via a split-intein base editing strategy.	CRISPR-Cas9 (HDR-iPSC) Base editing
[135]	ABCA4 c.4253+4C>T and c.3211_3212insGT—Therapeutic approach	Gene correction was gene editing via ssODN-mediated repair, targeting two specific mutations which was not replicated when TALEN was employed.	CRISPR-Cas9 (HDR-iPSC) and TALEN
[136]	ABCA4 c.4539+2001G>A variant in intron 30—Therapeutic approach	Gene correction of splicing defects and reduced pseudoexon-containing transcripts.	CRISPR-Cas9 (HDR-iPSC)
[137]	PROM1 c.1354dupT—Therapeutic approach	Gene correction achieved in 10% of cell lines.	CRISPR-Cas9 (HDR-iPSC)
[138]	CYP4V2 c.802-8_810del17insGC—Therapeutic approach	Gene correction restored BCD-iPSCs resistance to blue light damage.	CRISPR-Cas9 (HDR-iPSC)
[139]	TIMP3 610A>T—Therapeutic approach	Gene correction resulted in reduced basal laminar and sub-RPE calcium accumulation and improved oxidative stress response in SFD RPE.	CRISPR-Cas9 (HDR-iPSC)
[140]	ITM2B c.782A>C, p.G261A—Therapeutic approach	Successful gene correction for creation of isogenic control, confirmed using sanger sequencing.	CRISPR-Cas9 (HDR-iPSC)
[141]	CLCN2 c.2257C>T (p.R753X)—Therapeutic approach	Gene correction leading to rescue of chloride channel function and restoring outer segment phagocytosis.	CRISPR-Cas9 (HDR-iPSC)
[142]	AAVS1/PPP1R12C—Therapeutic approach	Knock-in Jaws EGFP, a chloride pump that renders cells sensitive to red light, into the iPSC genome at the AAVS1 locus.	CRISPR-Cas9 (HDR-iPSC)
[143]	CHM exon 1—Disease modeling	CHM knock -out to create an isogenic control and to create a disease model. Knock-out resulted in loss of REP-1, reduced pigmentation of iPSC RPE cells, defective Rab prenylation, increased mTORC1 signaling, and reduced autophagic flux.	CRISPR-Cas9 (NHEJ-iPSC)
[144]	(1) CHM c.809insC—Disease modeling	(1) Isogenic controls created for disease modeling by CHM knock-out from healthy iPSC	(1) CRISPR-Cas9 of knock-out (NHEJ-iPSC)
	(2) CHM c.808C>T—Therapeutic approach	(2) REP1 protein was found absent in CHM KO line and restored CHM+ iPSC after HDR correction. REP2 expression was unchanged in both lines. Both lines retained normal RPE morphology, normal phagocytosis. CHM KO RPE line was noted to have reduced pigmentation.	(2) CRISPR-Cas9 correction (HDR-iPSC)
[145]	CHM—Therapeutic approach	Introduced frameshift mutation in exon 6 of healthy iPSCs and hOMP RPE for disease modeling and then gene correction via CRISPR-Cas9. Exon 6 skipping produced a partially functional REP-1 protein that rescued phagocytic defects under oxidative stress, particularly via effects on RAB38 prenylation.	CRISPR-Cas9 (Exon skipping-iPSC and hOMP RPE)
[146]	NR2E3 c.119-2A>C and c.219G>C (p.R73S)—Therapeutic approach	Gene correction of patient iPSCs and restoration of wild-type NR2E3 transcript detected after 9 weeks in corrected retinal cells.	CRISPR-Cas9 (HDR-iPSC)
[147]	RS1 c.625C>T (p.R209C) and c.488G>A (p.W163)—Therapeutic approach	Knock-in of C625T mutation into healthy iPSCs and then using base editing for gene correction. Restoration of structural and molecular defects observed in 50% of patient-derived Ros.	CRISPR-Cas9 (HDR-iPSC) Base editing
[148]	RS1 c.C304T (p.R102W)—Disease modeling	Knock-in of c.C304T, p.R102W mutation into RS1gene of healthy iPSCs for disease modeling.	CRISPR-Cas9 (HDR-iPSC)
[149]	RS1 c.304C>T (p.R102W)—Therapeutic approach	To generate gene-corrected isogenic cell line from patient.	CRISPR-Cas9 (HDR-iPSC)
[150]	RS1 c.625C>T—Disease modeling	Knock -in of c.625C>T mutation into RS1 gene of healthy iPSCs for disease modeling.	Carboxylated nanodiamond-mediated CRISPR-Cas9 (HDR-iPSC)
[151]	OAT c.1205T>C—Therapeutic approach	Gene correction in patient iPSCs for further study. Correction restored OAT enzyme function, normalizing ornithine levels.	CRISPR-Cas9 (HDR-iPSC)
[152]	OA1 g.25288G>A—Therapeutic approach	Gene correction in patient iPSCs and rescue of normal GPR143 splicing and expression.	CRISPR-Cas12a (HDR-iPSC)
[153]	C1QTNF5 c.489C>G (p.S163R)—Therapeutic approach	Isogenic line generated and used as control. Upon RPE differentiation, edited line lost C1QTNF5 expression and presented increased phagocytosis when compared to control.	CRISPR-Cas9 (HDR-iPSC)
[154]	BEST1 p.I205T and p.Y236C—Therapeutic approach	Restored calcium currents in IPSC-RPE.	dCas9-KRAB-MeCP2 (iPSC-RPE)
[155]	RB1 c.374_380del (7 bp) and p.E125Vfs9—Disease modeling	When differentiated into ROs, RB1 knock-out cells showed a lack/reduced organization of retinal layers and inefficient differentiation of each retinal cell type but did have persistent proliferation of cone PRs.	CRISPR-Cas9 (NHEJ-hESC)
[156]	RB1 c.374_380del and p.E125Vfs9—Therapeutic approach	RB1-null cells are capable of forming large, neuronally enriched teratomas, potentially driven by ZEB1 activation, which also induces dysregulation of mitochondrial quantity and function; notably, these cells exhibit heightened sensitivity to carboplatin in drug screening assays.	CRISPR-Cas9 (NHEJ-hESC)
[157]	Cyp4v3 exon 1—Disease modeling	Cyp4v3 c.278_288del11bp knock-out mice exhibited hallmark features of Bietti crystalline dystrophy, including retinal crystalline deposits, RPE atrophy and degeneration, and reduced electroretinography (ERG) a- and b-wave amplitudes under both scotopic and photopic conditions. Additionally, the presence of crystalline deposits was found to be age-dependent.	CRISPR-Cas9 (NHEJ-animal ESC)
[158]	Cnga3 intron 5—Disease modeling	Cnga3 knock-in mouse model generated carries a 393 bp floxed miniSTOP cassette. STOP cassette did not lead to statistically significant reduction in b-wave amplitude when compared to both homozygous (Cnga3+/+) and heterozygous counterparts. Although a decrease in CNGA3 protein expression was observed in these models, cone electroretinographic (ERG) responses remained unaffected.	Easi-CRISPR (HDR-animal ESC)
[159]	NRL exon 2 c.221_222insAATTC (p.W74)—Disease modeling	Following differentiation into retinal organoids, NRL-deficient cell lines demonstrated diminished immunoreactivity for both NRL and NR2E3 at 14 weeks post-differentiation. These organoids failed to produce rod photoreceptors and instead exclusively generated S-cone–like photoreceptors.	CRISPR-Cas 9 (HDR-hESC)
[160]	RB1 exon 3—Disease modeling RB1 exon 1—Disease modeling	RB exon 3 c.374_380del (7-bp frameshift) and exon 1 whole-exon deletion (~400 bp) biallelic inactivation was confirmed by both immunohistochemistry and Western blot analysis. The resulting retinal organoids exhibited increased size, attributed to enhanced S-phase entry and cellular proliferation. RB1 loss was also associated with a reduced number of PRs, RGCs, and bipolar cells.	CRISPR-Cas 9 (NHEJ-hESC)
[161]	Rx gene exon 1—Disease modeling	Rx 41-base pair deletion was achieved. Differentiation into retinal organoids was associated with reduced Sox1 and Pax6 expression, loss of apical-basal polarity, and upregulation of Islet1/2, NFIA, HES5, and EBF1/3, indicating precocious differentiation. Wnt7b, Wnt8b, and R-spondin 4 were concurrently downregulated.	CRISPR-Cas 9 (NHEJ-hESC)
[162]	THRB exon 1—Disease modeling	ROs derived from the edited cells with an early translational termination in the first exon maintained an unchanged S-to-L/M cone ratio.	CRISPR-Cas9 (NHEJ-hESC)
[163]	CPAMD8 exon 4—Therapeutic approach	The homozygous frameshift null knock-out line generated showed normal morphology and pluripotency markers, with expected CPAMD8 truncation and dysfunction.	CRISPR-Cas9 (NHEJ-hESC)
[164]	LRP2 exon 2—Therapeutic approach	The homozygous LRP2 frameshift null knock-out generated retained typical pluripotent morphology and marker expression. Upon differentiation into RPE cells, displayed normal hexagonal morphology and expressed characteristic markers including ZO-1, MITF, and RPE65.	CRISPR-Cas9 (NHEJ-hESC)
[165]	BLIMP1 Exon 7—Disease modeling	3′ UTR EGFP insertion lead to BLMP1-GFP being detected throughout all stages of photoreceptor development, while PHLDA1 showed high expression in photoreceptor precursors and is linked to their temporal specification.	CRISPR-Cas9 (HDR-hESC)
[166]	GNGT2—Disease modeling	Biallelic T2A-mCherry knock-in strongly labels GNGT2-expressing cones throughout differentiation, enabling visualization of their significant migratory activity.	CRISPR-Cas9 (HDR-hESC)
[167]	WDR5B—Disease modeling	Coding sequence deletion (knock-out) and C-terminal 3XFLAG insertion (knock-in) were performed. Gene regulating WDR5B is only present in placental animals and is upregulated during neural differentiation. Knock-out of WDR5B causes increased cell death and dysfunctional proliferation in genetically altered cell lines that were differentiated into RPE cells.	CRISPR-Cas9 (NHEJ for knockout and HDR for knock-in-hESC)
[168]	NRL—Disease modeling	rGFP insertion lead to successful labelling of NRL+ rods during development.	CRISPR-Cas9 (HDR-hESC)
[169]	BRN3B—Disease modeling	Selective expression of tdTomato under the RGC-specific promoter insertion. This editing resulted in differentiated human RGCs which had a 96.5% expression of RGC marker RBPMS, confirming its similarity to innate RGCs. Tracking of soma, neurite development, and synaptic development was observed. HRGCs were also electrically responsive.	CRISPR-Cas9 (HDR-hESC)
[170]	BRN3B—Disease modeling	Insertion of a fluorescent reporter gene (tdTomato) and surface tag (Thy1.2) in BRN3B’s ORF allowed the tracking of over 150 microRNA when genetically engineered cells were differentiated into RGCs. Identification of miR-204-5p to be highly active in RGCs and miR-302b-3p to be highly active in ESCs.	CRISPR-Cas9 (HDR-hESC)
[171]	B2M 1-bp insertion and CIITA 1-bp deletion—Therapeutic approach	SKO-B2M cells attenuate CD8+ T cell activation, while DKO cells suppress both CD8+ and CD4+ T cell activation. However, edited cells elicit an enhanced natural killer (NK) cell response.	CRISPR-Cas9 (NHEJ-hESC)
[172]	BRN3B—Disease modeling	Knock-in of a P2A-tdTomato-P2A-THY1.2 reporter sequence at stop codon [173]. This model allowed for accurate visual identification of RGCs, improving differentiation, purification, and thereby mass scale production.	CRISPR-Cas9- (HDR-hESCs)
[174]	BRN3B—Disease modellingmodeling	Knock- in P2A-mCherry sequence into the 3′ end of the BRN3B ORF [173,175] which allows for tracking of differentiation to RGCs and identification of forskolin which can increase the percentage of RGCs generated from hESCs and identification of nanoscaffolds that guide axonal growth of RGCs.	CRISPR Cas 9 (HDR-hESC)
[173]	AAVS1—Disease modeling	BEST1 promoter and EGFP insertion enabled tracking of differentiation of this hESC line into RPE cells which were of normal karyotype, expressed pluripotency markers, and were able to differentiate.	CRISPR-Cas9 (HDR-hESC)
[176]	Sox2, Klf4 and Oct4—Disease modeling	The editing used hyperdCas12a to simultaneously activate Oct4, Sox2, and Klf4 in retinal progenitor cells, reprogramming their differentiation in vivo.	dCas12a Multiplex activation (HEK293T and Intravitreal murine retina)
[175]	CRX—Disease modeling	Targeted cleavage at the CRX locus to enable knock-in of the GFP reporter allowed for accurate and valid tracking of PR precursors which enabled the study of PR development. No off-target mutations were noted.	ZFN (GFP insertion-hESC)
[177]	RAX—Disease modeling	RAX-targeted insertion of GFP enabled fluorescent tracking of retinal progenitor cells derived from rat ESCs, facilitating the development and transplantation of neuron-enriched RPCs that significantly preserved vision in a rat model of retinal degeneration.	TALEN (GFP insertion-Animal ESC)

## Data Availability

No new data were created or analyzed in this study.

## Abbreviations

AAV	Adeno-associated virus
AAVS1	Adeno-associated virus integration site 1
ABE	Adenine base editor
ABCA4	ATP-binding cassette subfamily A member 4
ADRP	Autosomal dominant retinitis pigmentosa
AMD	Age-related macular degeneration
AIPL1	Aryl-hydrocarbon-interacting protein-like 1
ARRP	Autosomal recessive retinitis pigmentosa
BCVA	Best-corrected visual acuity
BD	Best disease
BEST1	Bestrophin-1
BVMD	Best vitelliform macular dystrophy
C3	Complement component 3
Cas9	CRISPR-associated protein 9
CBEs	Cytosine base editors
CEP290	Centrosomal protein 290
CFH	Complement factor H
CHM	Choroideremia gene (Rab escort protein 1 coding gene)
CLCN2	Chloride voltage-gated channel 2
CPPs	Cell-penetrating peptides
CRB1	Crumbs homolog 1
CRISPR	Clustered Regularly Interspaced Short Palindromic Repeats
CRISPRa	CRISPR activation
CRISPRi	CRISPR interference
CRX	Cone-rod homeobox
DSB	Double-strand break
EGFP	Enhanced green fluorescent protein
EOMD	Early-onset macular drusen
ESCs	Embryonic stem cells
ESCS	Enhanced S-cone syndrome
EYS	Eyes shut homolog
FHL-1	Factor H-like protein 1
gRNA	Guide RNA
GWAS	Genome-wide association study
HDR	Homology-directed repair
hESCs	Human embryonic stem cells
hiPSCs	Human induced pluripotent stem cells
HITI	Homology-independent targeted integration
HLA	Human leukocyte antigen
hPSCs	Human pluripotent stem cells
IRDs	Inherited retinal dystrophies
iPSCs	Induced pluripotent stem cells
IVS26	Intron variant sequence 26 (CEP290 deep-intronic mutation)
KO	Knockout
L-ORD	Late-onset retinal degeneration
LCA	Leber congenital amaurosis
LCA2	Leber congenital amaurosis type 2
LCA4	Leber congenital amaurosis type 4
LCA5	Leber congenital amaurosis type 5
LCA7	Leber congenital amaurosis type 7
LNPs	Lipid nanoparticles
LRP2	Low-density lipoprotein receptor-related protein 2
MERTK	MER proto-oncogene, tyrosine kinase
MMEJ	Microhomology-mediated end joining
MSCs	Mesenchymal stem cells
mRNA	Messenger ribonucleic acid
nCas9	Cas9 nickase
NHEJ	Non-homologous end joining
NR2E3	Nuclear receptor subfamily 2 group E member 3
OA1	Ocular albinism type 1
OAT	Ornithine aminotransferase
PAM	Protospacer adjacent motif
pegRNA	Prime editing guide RNA
PDE6	Phosphodiesterase 6
POS	Photoreceptor outer segments
PR	Photoreceptors
PRPH2	Peripherin-2
PROM1	Prominin-1
RGCs	Retinal ganglion cells
REP1	Rab escort protein 1
RHO	Rhodopsin
RNP	Ribonucleoprotein
ROs	Retinal organoids
RPCs	Retinal progenitor cells
RP	Retinitis pigmentosa
RP2	Retinitis pigmentosa 2 protein
RP13	Retinitis pigmentosa type 13
RPGR	Retinitis pigmentosa GTPase regulator
RPE	Retinal pigment epithelium
RPE65	Retinal pigment epithelium-specific protein 65 kDa
RS1	Retinoschisin 1
RT	Reverse transcriptase
SFD	Sorsby fundus dystrophy
sgRNA	Single-guide RNA
SNVs	Single-nucleotide variants
SNCs	Silica nanocapsules
ssODN	Single-stranded oligodeoxynucleotide
STGD1	Stargardt disease type 1
TALENs	Transcription activator-like effector nucleases
TP53	Tumor protein p53
UPR	Unfolded protein response
VSX2	Visual system homeobox 2
WT	Wild type
XLRS	X-linked juvenile retinoschisis
XLRP	X-linked retinitis pigmentosa
ZFNs	Zinc finger nucleases
